# Competing value signals impair reward-learning via dopaminergic mechanisms and increase exploration

**DOI:** 10.1371/journal.pbio.3003922

**Published:** 2026-07-24

**Authors:** Wen-Wei Lin, Pei-Yu Lee, Hsin-Yun Tsai, Yi-Hsuan Lin, Min-Min Lin, Zheng-Liang Lu, Mei-Yu Yeh, Ming-Tsung Tseng

**Affiliations:** 1 Graduate Institute of Brain and Mind Sciences, National Taiwan University College of Medicine, Taipei, Taiwan; 2 Taiwan International Graduate Program in Interdisciplinary Neuroscience, National Taiwan University and Academia Sinica, Taipei, Taiwan; 3 Department of Computer Science and Information Engineering, National Taiwan University, Taipei, Taiwan; Oxford University, UNITED KINGDOM OF GREAT BRITAIN AND NORTHERN IRELAND

## Abstract

Effective reinforcement learning requires balancing exploration of uncertain options with exploitation of known outcomes. In real-world contexts, the same action may yield rewards in some situations and punishments in others, yet how these learning processes influence each other remains unclear. Here, we examine the neural mechanisms underlying how reward and punishment learning interact to guide adaptive behavior. We conducted four experiments (*N* = 159) using an instrumental learning task with binary choices, some of which were exclusive to reward or punishment learning trials, while others appeared in both, allowing assessment of their interaction. When choices were tied to a single learning type, reward learning engages less exploration (i.e., fewer choices of lower-value options) than punishment learning. Critically, when both learning processes were concurrently engaged, reward learning was selectively impaired, accompanied by enhanced exploration and greater activation in exploration-related prefrontal regions revealed by fMRI. Computational modeling showed that impaired reward learning was best explained by sensitivity to prior punishment history associated with reward-learning options, while individual differences in loss aversion predicted the degree of increased exploration. Finally, pharmacological attenuation of dopaminergic signaling via the D2/3 receptor antagonist amisulpride abolished both the increased exploration and the interference with reward learning. These findings suggest that punishment-history interference during reward learning is dopamine-modulated and associated with increased exploration, with individual differences in this exploration linked to loss aversion, providing a mechanistic account of how the brain resolves competing value signals and informing dopamine-related learning disturbances in neuropsychiatric conditions.

## Abstract 2

How do learning processes related to reward exploitation and exploration interact with one another? This study uses behavior, fMRI, and pharmacology to show that sensitivity to punishment histories biases reward learning, increasing exploration and reducing reward maximization via dopaminergic mechanisms, with individual differences linked to loss aversion.

## Introduction

Maximizing rewards and minimizing punishments are fundamental to adaptive behavior. However, in real-world contexts, actions are often influenced by both reward and punishment learning histories. For example, biking to work may be reinforced by prior time-saving experiences (reward) but also discouraged by previous instances of getting soaked on rainy days (punishment). Prioritizing rewards over punishments may lead individuals to bike even on cloudy days, undermining punishment avoidance, whereas prioritizing punishment avoidance may reduce biking even on clear days, limiting reward opportunities. In such scenarios, optimal decisions depend on the interaction between reward- and punishment-driven learning. Although animal studies have examined this interplay [1,2], evidence in humans remains limited. Human research has typically examined reward and punishment learning in isolation using independent trial types [3–8], or employed bivalent cues that concurrently signal both reward and punishment outcomes within a single trial [9–11]. However, neither approach permits direct investigation of how reward and punishment learning interact in decisions where both are simultaneously relevant, leaving it unclear how the brain integrates competing value signals in such contexts.

A salient attribute that may underlie this interaction is valence. Evidence indicates a valence-dependent asymmetry in processing positive and negative information [12–14], which is informed by two primary theoretical perspectives. The first, positivity bias, posits that positive information generally receives preferential processing [15–17]. In learning tasks, prior work has documented that behavior can be more strongly influenced by positive than by negative outcomes [18–20]. The second perspective, loss aversion, emphasizes the dominance of negative information [21–23], with individuals weighting negative outcomes more heavily than equivalent positive ones [24–26]. This negativity bias has been shown to influence cognitive performance [27], including learning behaviors [7]. Together, these biases may arguably shape the interplay between reward and punishment learning, though the precise role of valence in these interactions remains to be clarified.

If valence-dependent asymmetry plays a role, a critical question arises: How does valence influence choice behavior during reinforcement learning? In reinforcement learning, individuals balance exploration of uncertain options with exploitation of predictable ones to associate behaviors with consequences, where an effective balance supports adaptive decisions [28–30]. Exploration, in particular, is modulated by dopaminergic signaling, affecting how individuals adjust their behavior when evaluating alternative options [31–34]. Notably, research indicates that negative outcomes prompt more exploratory behavior than positive outcomes [29,35–37]. In value-based decision-making, exploratory tendencies can be expressed as deviations from value-guided choice [29,31,38–43]. This valence asymmetry therefore gives rise to a compelling hypothesis: while engaged in a reward learning task in which the reinforcement history of a choice is also related to punishment learning, such associations may be linked to reduced reward maximization [44,45], as reflected in increased exploration, particularly in loss-averse individuals.

The present study aimed to investigate how reward and punishment learning interact to support adaptive behavior. To test the hypothesis described above, we conducted Experiments 1 and 2 (fMRI studies), where participants performed a probabilistic instrumental learning task involving binary choices, some linked to both reward and punishment learning. Both experiments showed that punishment learning interfered with reward learning. We then applied computational modeling to confirm this interference and to examine whether it was associated with increased exploration following punishment learning history, analyzing both exploration rates and fMRI BOLD signals. Across Experiments 1 and 2, increased exploration and its neural correlates were associated with the observed interference. To assess whether this interference and exploration increase were linked to loss aversion, Experiment 3 employed a similar task with varying monetary gains and losses and included an independent measure of loss aversion. Both model-free and model-based analyses implicated loss aversion in the interference, with individual loss aversion predicting exploration. Finally, Experiment 4 examined dopaminergic involvement using a dopamine antagonist, which abolished both enhanced exploration and the associated reward-learning interference. These findings provide novel insight into how the brain resolves competing value signals and highlight dopamine’s role in modulating exploration to support adaptive choice.

## Results

### Experiments 1 and 2: Enhanced exploration in reward-learning interference by punishment learning

In Experiments 1 and 2, participants were excluded if their average correct rate across the two control conditions fell below chance level (50%) (see Materials and methods, Participants). After applying this criterion, the final sample sizes were: Experiment 1: *N* = 30; Experiment 2: *N* = 30.

#### Reward-learning interference by punishment learning.

**Fig 1:**
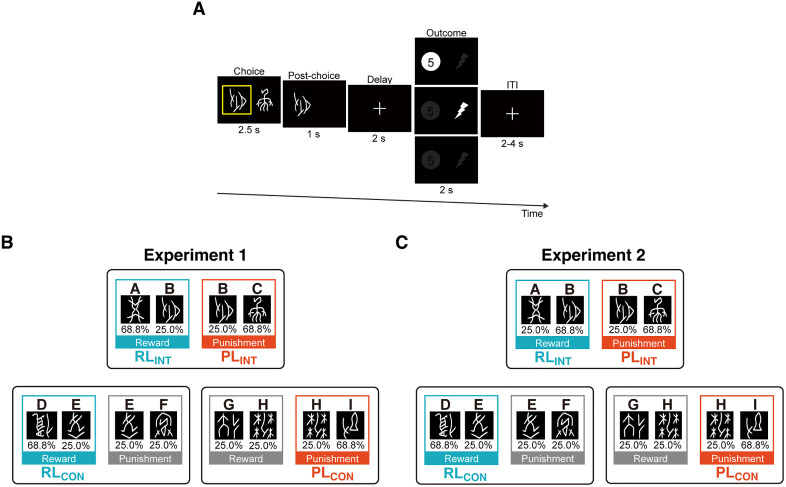
Experimental design. **(A**) Trial structure of the learning task. Each trial began with a 2.5 s presentation of an option pair (oracle bone scripts). After selection, the chosen option remained on screen for 1 s, followed by a 2 s delay. The outcome phase lasted 2 s, delivering either a reward (5 TWD), a punishment (painful stimulation in the pain-related task or monetary loss in the monetary loss-related task), or nothing. **(B)** Probability composition of the six option pairs in Experiment 1, grouped into three clusters of interrelated pairs (black frames). These included two asymmetric reward-learning pairs (blue: *AB* pair for the reward-learning interference condition, RL_INT_; *DE* pair for the reward-learning control condition, RL_CON_), two asymmetric punishment-learning pairs (red: *BC* pair for the punishment-learning interference condition, PL_INT_; *HI* pair for the punishment-learning control condition, PL_CON_), and two symmetric pairs (gray: *EF* and *GH* pairs). The percentage below each oracle bone script indicates the probability of reward or punishment. **(C)** The probability composition in Experiment 2 is basically the same as in Experiment 1 (Fig 1B), except that the probability of A and B was reversed.

To first investigate whether the simultaneous involvement of reward- or punishment-related options interfered with learning performance, we designed Experiment 1, in which healthy volunteers performed a probabilistic instrumental learning task with binary choices (Fig 1A). On each trial, participants chose between two options (oracle bone scripts), with each option associated with a specific probability of reward (5 New Taiwan dollars, TWD) or punishment (monetary loss or painful stimulation). To control for incentive motivation, we equated punishments to the reward of 5 TWD (see Materials and methods, Experimental design, *Incentive calibration* for details). A total of six option pairs were used, grouped into three interrelated clusters via overlapping options (*AB* and *BC*, *DE* and *EF*, and *GH* and *HI*; Fig 1B). Pairs with unequal probabilities (68.8/25.0%) of rewards (*AB* and *DE* pairs) and punishments (*BC* and *HI* pairs) were designed to respectively encourage reward-seeking or punishment avoidance, with learning required for successful performance [3,5,46,47]. Central to the design, the *AB* pair served as the reward-learning interference condition (designated as RL_INT_), in which a choice option (i.e., *B*) also appeared in a punishment learning condition (i.e., *BC* pair); likewise, the *BC* pair served as the punishment-learning interference condition (designated as PL_INT_). To establish control conditions, pairs with symmetrical reward (*GH* pair) or punishment (*EF* pair) probability pairs (25.0/25.0%) were included [8,10,48]. Compared with the symmetric *EF* pair, the *BC* pair had asymmetrical outcome probabilities and a higher overall punishment rate, enabling us to test whether reward learning in the *AB* pair—relative to the *DE* pair—was hindered by punishment-avoidance tendencies. Likewise, compared with the symmetric *GH* pair (25% reward for each option), the *AB* pair’s asymmetrical probabilities and higher reward rate allowed us to examine whether punishment learning in the *BC* pair—relative to the *HI* pair—was biased by a preference for reward outcomes (see Materials and methods, Experimental design, *Learning task* for detailed rationale). Thus, the *DE* pair (reward-learning control condition, designated as RL_CON_) served as the control for the *AB* pair (RL_INT_), and the contrast between RL_CON_ and RL_INT_ trials allowed assessment of punishment learning’s impact on reward learning. Similarly, the *HI* pair (punishment-learning control condition, designated as PL_CON_) served as the control for the *BC* pair (PL_INT_), and the contrast between PL_CON_ versus PL_INT_ trials represented reward learning’s impact on punishment learning. Across trials, participants were randomly presented with one of the six pairs and learned to select the best option of each pair (i.e., highest reward or lowest punishment probability).

**Fig 2:**
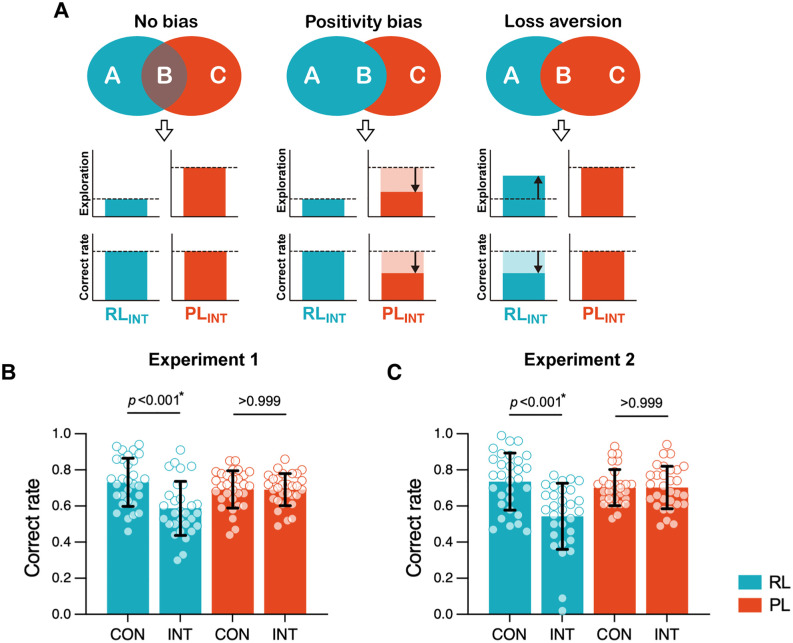
Predicted behavioral patterns and observed interference of reward learning performance by punishment learning in Experiments 1 and 2. **(A)** Conceptual illustration of task-specific interactions between reward and punishment learning in trials with an overlapping option (reward-learning interference, RL_INT_; punishment-learning interference, PL_INT_). The schematic illustrates predicted patterns under different theoretical assumptions (see Introduction). Left panel: If the two learning processes operate independently (i.e., no bias; overlapping area shown in mixed blue and red), RL_INT_ and PL_INT_ performance should resemble their respective controls (reward-learning control, RL_CON_; punishment-learning control, PL_CON_). Consistent with prior work (see Introduction), reward learning (blue exploration bar) generally favors exploitation, whereas punishment learning (red exploration bar) entails greater exploration in this task. Middle panel: Under a positivity bias (overlapping area in blue), prior reward history of the overlapping options (e.g., *B* in *AB*) leads individuals to prioritize reward seeking over punishment avoidance, resulting in reduced exploration and impaired task performance in PL_INT_ trials (punishment-minimization failure). Right panel: Under a loss-aversion bias (overlapping area in red), prior punishment history of the overlapping options (e.g., *B* in *BC*) leads individuals to prioritize punishment avoidance over reward seeking, resulting in enhanced exploration and impaired performance in RL_INT_ trials (reward-maximization failure). **(B)** In Experiment 1, correct rates were lower in RL_INT_ than RL_CON_ trials (*p* < 0001; monetary loss and pain pooled), indicating reward-learning interference by punishment learning. No difference was found between PL_INT_ than PL_CON_ trials (*p* > 0.999). **(C)** Experiment 2 showed similar results, with lower correct rates in RL_INT_ than RL_CON_ trials (*p* < 0001; monetary loss and pain pooled) and no difference between PL_INT_ than PL_CON_ trials (*p* > 0.999). All *p* values are Bonferroni-corrected in repeated-measures ANOVAs. Data are represented as mean ± SD. The data underlying Fig 2B and 2C can be found at https://doi.org/10.17605/OSF.IO/T7YWA.

Fig 2A presents the predicted patterns of task performance and exploratory behavior based on proposed theoretical perspectives. The first pattern (no bias) suggests no interaction between reward and punishment learning. Based on previous studies (see Introduction), reward learning favors exploitation (low exploration), while punishment learning entails greater exploration. The other two patterns reflect valence-dependent asymmetry in information processing, suggesting that choices simultaneously associated with both reward and punishment learning (i.e., RL_INT_ and PL_INT_ trials) are affected by the interrelated pair. Under a positivity bias, reward-associated pairs exert a stronger influence on subsequent choices. When *B* had previously appeared in a reward-learning context, participants were more likely to exploit its perceived value, reducing the exploration necessary for acquiring the punishment contingency in *BC*. This under-exploration impaired PL_INT_ performance. In contrast, under loss aversion, associating a reward-learning choice (*B* in *AB* pair) with punishment learning (*BC* pair) increased exploration, impairing reward learning performance in RL_INT_ trials relative to RL_CON_ trials.

For self-reported incentive ratings, a 2 (motivation: pursuing rewards or avoiding punishments) × 2 (punishment type: monetary loss or pain) repeated-measures ANOVA revealed neither significant main effects nor significant interaction (all *p* ≥ 0.103). Post hoc comparisons confirmed no significant differences in incentive ratings between reward pursuit and punishment avoidance, regardless of whether the punishment was monetary loss or pain (both *p* > 0.999). These results suggest that participants exhibited comparable motivation for both reward pursuit and punishment avoidance. As expected, the correct rates for the two control conditions (RL_CON_ and PL_CON_) were significantly above chance (i.e., 50%; all *p* < 0.001), confirming successful instrumental learning (S1 Table).

Because punishment type (monetary loss versus pain) yielded no significant main effects or interactions in Experiments 1 and 2, as assessed in a 2 (learning type: reward or punishment learning) × 2 (interference type: control or interference condition) × 2 (punishment type: monetary loss or pain) repeated-measures ANOVA (S1 Table), data were pooled for subsequent analyses. Pooling did not alter the interference effect observed in the full model.

To examine whether punishment learning-related choices influenced reward learning (or vice versa), we then performed a 2 (learning type) × 2 (interference type) repeated-measures ANOVA on the pooled data. This analysis showed no significant main effect of learning type (*p* = 0.151), but a significant main effect of interference type (*F*_1,29_ = 16.4, *p* < 0.001). Of note, there was a significant learning-by-interference interaction (*F*_1,29_ = 15.8, *p* < 0.001), suggesting that the impact of interference varied across learning types. Post hoc comparisons demonstrated that RL_INT_ trials had significantly lower correct rates than RL_CON_ trials (*p* < 0.001), while PL_CON_ versus PL_INT_ differences were not significant (*p* > 0.999; Fig 2B and S1 Table).

Although the finding from Experiment 1 that punishment associations interfered with reward learning could be explained by participants’ loss aversion (Fig 2A), it may also stem from contextual differences in the overlapping option (*B*), which was the worst in reward learning and the best in punishment learning (Note: in the present study, “contextual effect” refers specifically to relative value comparisons within choice pairs, acknowledging varied definitions in the literature). Previous studies suggest that option values are computed relative to the alternative within the same choice pair; the option yielding a more favorable outcome is typically preferred, regardless of whether the outcomes are framed as rewards or punishments [5,47,49]. To differentiate between these explanations, we designed Experiment 2, altering the context of the overlapping *B* option to be the better choice in both *AB* and *BC* pairs (Fig 1C). If the reward-learning interference observed in Experiment 1 was context-driven, the correct rate in RL_INT_ trials should not worsen relative to RL_CON_ trials in Experiment 2. However, if loss aversion underlay the interference, we expected the same interference pattern as in Experiment 1.

Similar to Experiment 1, participants in Experiment 2 showed no significant differences in incentive ratings between pursuing rewards or avoiding punishments (both *p* ≥ 0.889). RL_CON_ and PL_CON_ correct rates were both significantly above chance (both *p* < 0.001), confirming successful learning (S1 Table).

The interference pattern in Experiment 1 was replicated. The two-way repeated-measures ANOVA described above again revealed a significant learning-by-interference interaction (*F*_1,29_ = 13.8, *p* < 0.001). Post hoc comparisons revealed that RL_INT_ trials had significantly lower correct rates than RL_CON_ trials (*p* < 0.001), while the correct rate in PL_CON_ and PL_INT_ trials was similar (*p* > 0.999; Fig 2C and see S1 Table for full 2 × 2 × 2 data and the three-way repeated-measures ANOVA).

The interference effect observed in Experiments 1 and 2 persisted regardless of whether participant exclusion was based on average performance across RL_CON_ and PL_CON_ (current criterion) or on performance in either condition (learning-by-interference interaction: both *p* < 0.001; Post hoc correct-rate comparisons: RL_INT_ versus RL_CON_, both *p* < 0.001; PL_INT_ versus PL_CON_, both *p* ≥ 0.981). A 2 (learning type) × 2 (interference type) × 2 (experiment: Experiment 1 or 2) repeated-measures ANOVA showed no significant three-way interaction (*p* = 0.436), suggesting that reversing the probability composition of *A* and *B* options in the *AB* pair did not significantly impact punishment learning’s interference with reward learning.

#### Computational modeling.

After ruling out contextual effects as the cause of reward-learning interference from punishment learning, we employed computational modeling to test whether this interference stemmed from prior punishment learning acquired in the interrelated pair. We first examined three models that combined a delta learning rule with a softmax choice rule (see Materials and methods, Behavioral analysis and computational modeling of the learning task). The first model (Perseveration model, i.e., Model 5 in S2 Table) included a perseveration term to capture value-independent choice-repetition effects, a phenomenon well documented in prior reinforcement-learning and exploration research [31,50–52]. Because we hypothesized that a punishment-associated history for a reward-learning option may impair reward maximization, the second model (Perseveration + Interference at Choice model, i.e., Model 6 in S2 Table) was identical to the Perseveration model except that the choice rule incorporated an interference term (*S*), which captures the influence of punishment outcomes from the interrelated pair. The third model (Perseveration + Context model, i.e., Model 11 in S2 Table) was included to rule out contextual influences, paralleling the approach used in Experiment 2. This model matched the Perseveration model but additionally incorporated choice-context value, such that each option’s prediction error was adjusted by the pair’s context value, which served as a reference point for outcome evaluation [5,53]. To enhance parameter estimation reliability, we employed a hierarchical modeling approach [54]. Model performance was evaluated using the Watanabe–Akaike Information Criterion (WAIC) [55,56], which we used to assess each model’s goodness of fit. The reliability of our modeling procedure was examined through model- and parameter-recovery analyses based on simulated datasets (S1 and S2 Figs) [57].

**Fig 3:**
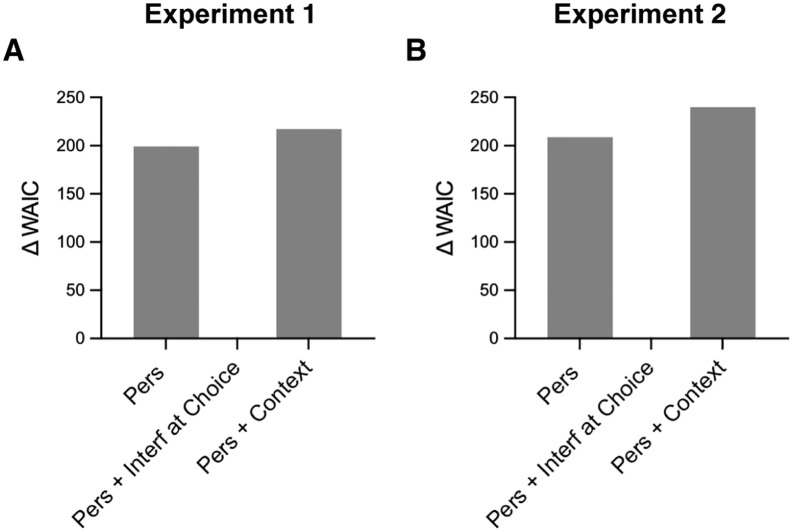
Model comparison. The figure shows Watanabe–Akaike Information Criterion (WAIC) differences relative to the best-fitting Perseveration (Pers) + Interference (Interf) at Choice model, with smaller values indicating a better fit. The data underlying this Figure can be found at https://doi.org/10.17605/OSF.IO/T7YWA.

Model comparison revealed that the Perseveration + Interference at Choice model (Model 6) provided the best fit for both Experiments 1 and 2 (Fig 3). Posterior predictive simulations confirmed that this model reproduced observed behavioral patterns (S3 Fig) and trial-by-trial learning dynamics across conditions (S4 Fig). These results indicate that, even after accounting for value-independent repetition, interference arising from punishment-learning history remains a key mechanism underlying the observed reward-learning interference. They also confirm that contextual effects do not account for this interference, consistent with the model-free results from Experiment 2.

Given that the main behavioral effect observed was reduced performance in RL_INT_ relative to RL_CON_ trials, we next examined how this effect was captured within the best-fitting Model 6. In this model, however, condition differences are evaluated indirectly from independently estimated condition-specific parameters, which introduces greater uncertainty in between-condition comparisons. To provide a more interpretable parameterization for evaluating condition differences in model parameters, we additionally implemented a baseline-referenced reparameterization of Model 6, in which the RL_CON_ parameter serves as the reference baseline and condition effects are expressed as deviations (e.g., RL_INT_, RL_CON_ + $\Delta S_{{RL}_{INT}}$; see Materials and methods). This reparameterization does not introduce a new computational hypothesis, but instead reformulates the original model using a baseline-referenced parameter structure. Relative to Model 6, the baseline-referenced Model 6 yielded highly similar model fit (S5 Fig) and behavioral predictions (S6 Fig).

As shown in S7 Fig and S3 Table, the baseline-referenced Model 6 revealed condition-dependent modulation of the interference parameter (*S*) at the level of group-level mean parameters (*μ*), although these effects varied across experiments and sessions. In Experiment 1, substantial directional evidence for condition differences (posterior probability > 0.90) was observed in one session, whereas two sessions showed such evidence in Experiment 2. Across these sessions, *S* tended to be lower in RL_INT_ than in RL_CON_, suggesting modulation by punishment-learning history in RL_INT_. In contrast, no session across experiments showed posterior probability > 0.90 for condition differences in the natural logarithm of the inverse temperature parameter (*β*) or perseveration parameter (*ρ*). Because the baseline-referenced Model 6 also yielded highly similar findings in subsequent analyses of exploration (S8 Fig), loss aversion (S9 Fig), and pharmacological manipulation (S10 Fig), Model 6 was retained for subsequent behavioral and neuroimaging analyses, including fMRI regressors.

In addition to the three primary models, we examined 16 additional combinations of perseveration, interference, and context value (Models 1–4 and 7–10, and 12), as well as variants examining cross-pair value generalization (Model 1a), asymmetric learning rates (Model 1b), and extended forms of perseveration (Models 5a–5c, 6a, 6b; see Materials and methods). None outperformed Model 6 (S2 Table).

#### Enhanced exploration during reward-learning interference.

Using the winning model, we then analyzed the relationship between individual exploration rates (the proportion of exploration trials) and task performance across participants. In this study, exploration was defined with respect to model-based expected values: trials were classified as exploratory if participants chose an option with a suboptimal expected value, and exploitative if they selected the option with the highest expected reward or lowest expected punishment [29,31,38–43] (see Materials and methods, Behavioral analysis and computational modeling of the learning task). This definition captures deviations from value-maximizing behavior based on subjective value estimates.

**Fig 4:**
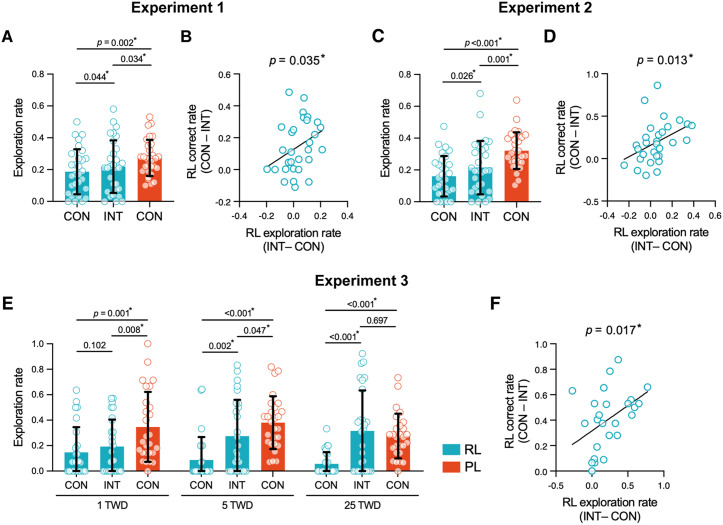
Relationship between reward-learning interference and exploration. **(A, B)** In Experiment 1, the exploration rate in punishment-learning control (PL_CON_) trials was significantly higher than that in reward-learning control (RL_CON_) trials (*p* = 0.002, one-tailed), and so was the exploration rate in reward-learning interference (RL_INT_) trials (*p* = 0.044, one-tailed). The increase in exploration during RL_INT_ trials relative to RL_CON_ trials was significantly correlated with a reduction in correct responses (*r* = 0.335, *p* = 0.035, one-tailed), indicating a link between heightened exploration and reward-learning interference. **(C, D)** In Experiment 2, the exploration rate pattern across RL_CON_, RL_INT_, and PL_CON_ trials replicated that of Experiment 1, and a significant relationship between exploration rates and correct rates in RL_INT_ vs. RL_CON_ trials was observed (*r* = 0.405, *p* = 0.013, one-tailed). **(E, F)** In Experiment 3, PL_CON_ trials showed consistently higher exploration than RL_CON_ across all monetary amounts (all *p* ≤ 0.001). Except for the 1 TWD condition, where reward-learning interference was not significant (see Results for Experiment 3), the exploration rate in RL_INT_ trials was significantly higher than in RL_CON_ trials for the 5 and 25 TWD conditions (both *p* ≤ 0.002). Increased exploration in RL_INT_ trials significantly correlated with decreased correct rates compared to RL_CON_ trials (*r* = 0.411, *p* = 0.017, one-tailed; pooled across the 5 and 25 TWD conditions). In **(A)**, **(C)**, and **(E)**, all *p* values are Bonferroni-corrected in repeated-measures ANOVAs. Data are represented as mean ± SD. The data underlying Fig 4A, 4C, and 4E can be found at https://doi.org/10.17605/OSF.IO/T7YWA.

As shown in Fig 4A and S4 Table, participants in Experiment 1 exhibited significantly higher exploration rates in PL_CON_ relative to RL_CON_ trials (*p* = 0.002, one-tailed), and in RL_INT_ relative to RL_CON_ trials (*p* = 0.044, one-tailed). Crucially, the increased exploration rates in RL_INT_ relative to RL_CON_ trials significantly covaried with reward-learning interference (*r* = 0.335, *p* = 0.035, one-tailed; Fig 4B). These findings were replicated in Experiment 2 (PL_CON_ versus RL_CON_: *p* < 0.001; RL_INT_ versus RL_CON_: *p* = 0.026; Fig 4C; correlation with interference: *r* = 0.405, *p* = 0.013; Fig 4D). Note that in Experiments 1 and 2, correct rates did not differ significantly between RL_CON_ and PL_CON_ trials (both *p* ≥ 0.268; Fig 2B and 2C), despite significantly higher exploration rates in PL_CON_ than RL_CON_ trials (Fig 4A and 4C). Moreover, although the correlation between increased exploration and reduced correct rates in PL_INT_ relative to PL_CON_ trials was significant in Experiment 1 (*r* = 0.45, *p* = 0.035), this relationship was not observed in Experiment 2 (*p* = 0.622). Taken together, these results indicate that higher exploration rates are associated with greater reward-learning interference, highlighting a behavioral link between exploration and task performance.

#### Brain responses to exploration during reward-learning interference.

In parallel with our behavioral findings, we next analyzed the fMRI data from Experiments 1 and 2 to further establish the neural link between exploration and reward-learning interference. By implementing trial-by-trial prediction error (i.e., the actual outcome minus expected outcome based on the winning model) as parametric modulators during the outcome period (general linear model 1, GLM1; see Materials and methods, Imaging data acquisition and analysis), we found that the BOLD signal within the ventral striatum (VS) significantly covaried with prediction error in both Experiment 1 (right VS: peak MNI = 14/12/ − 6, *t* = 4.93, *k* = 16, *p* = 0.001; left VS: peak MNI = −10/12/ − 4, *t* = 3.55, *k* = 2, *p* = 0.023; voxel-wise small-volume family-wise error corrected; S11A Fig) and Experiment 2 (right VS: peak MNI = 16/8/ − 6, *t* = 4.77, *k* = 25, *p* = 0.002; left VS: peak MNI = −10/14/ − 6, *t* = 3.77, *k* = 1, *p* = 0.020; voxel-wise small-volume family-wise error corrected; S11B Fig). This finding aligns with prior reinforcement learning studies [46,47,58–61].

**Fig 5:**
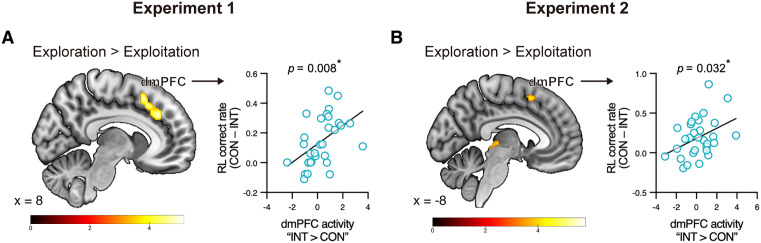
Relationship between reward-learning interference and exploration-associated neural responses in Experiments 1 and 2. Using general linear model (GLM) 2 (see Materials and methods), dorsomedial prefrontal cortex (dmPFC) exhibited significant activations during exploration (contrast “Exploration> Exploitation”). Activated clusters were corrected for multiple comparisons at the whole-brain level (cluster-forming threshold of *p* < 0.001, and a cluster-level familywise error rate of *p* < 0.05). The color bar shows SPM-derived *t* scores. See S5 Table for details. Based on GLM3 (see Materials and methods), the BOLD signal extracted from the suprathreshold dmPFC clusters significantly covaried with the reduction in correct rates in reward-learning interference (RL_INT_) vs. reward-learning control (RL_CON_) trials (Experiment 1: *r* = 0.476, *p* = 0.008; Experiment 2: *r* = 0.392, *p* = 0.032).

We then examined whether exploration-relevant brain structures indeed supported processes related to the reward-learning interference phenomenon. For this purpose, we first performed whole-brain analyses to search for the neural correlate that gave rise to exploration by comparing brain activity for exploratory trials against exploitative trials (i.e., contrast “Exploration> Exploitation” pooled over the two types of punishment) during the choice period (GLM2; see Materials and methods, Imaging data acquisition and analysis). This analysis revealed significant activation within the dorsomedial prefrontal cortex (dmPFC, encompassing the supplementary motor area and middle cingulate cortex) in both Experiment 1 (peak MNI = 8/16/50, *t* = 5.21, *k* = 394; Fig 5A) and Experimen*t* 2 (peak MNI = −8/12/52, *t* = 4.56, *k* = 162; Fig 5B; S5 Table), which have been repor*t*ed in previous studies on exploration [31,62,63].

Subsequently, we assessed whether the revealed dmPFC responses between RL_INT_ and RL_CON_ trials were related to the reward-learning interference phenomenon (GLM3; see Materials and methods, Imaging data acquisition and analysis). In line with the behavioral results (Fig 4B and 4D), there existed a linear relationship between the extent of reward-learning interference and the BOLD signal within the dmPFC (contrast “Exploration> Exploitation”) in both Experiment 1 (*r* = 0.476, *p* = 0.008; Fig 5A) and Experiment 2 (*r* = 0.392, *p* = 0.032; Fig 5B); that is, as dmPFC activation became stronger, the extent of interference increased linearly. By contrast, this linear relationship was not observed between dmPFC activation and the difference in correct rates between PL_CON_ and PL_INT_ trials in both experiments (both *p* ≥ 0.838).

### Experiment 3: Loss aversion in reward-learning interference by punishment learning

The above findings suggest that reward-learning interference associated with prior punishment history was accompanied by enhanced exploration. To further examine whether reward-learning interference (i.e., impaired task performance) and enhanced exploration were related to individual loss aversion, we conducted Experiment 3 (*N* = 27; after applying the same exclusion criterion as in Experiments 1 and 2), which included a learning task (identical to Experiment 2, but using only monetary losses as punishment, as Experiments 1 and 2 found no significant differences between monetary loss and pain) and a loss aversion task, presented in counterbalanced order (see Materials and methods, Experimental design).

We used two strategies to investigate the role of loss aversion. First, in the learning task, we introduced three levels of monetary gains and losses (1, 5, and 25 TWD, counterbalanced across three sessions, with each session using a single monetary amount), as prior evidence suggests that loss aversion depends on magnitude [64,65] and exerts stronger cognitive effects with larger potential losses [27]. If loss aversion accounted for the reward-learning interference, we anticipated greater monetary losses to be associated with larger increases in exploration and/or greater reductions in correct rates. Second, in the loss aversion task, adapted from Tom and colleagues [26], we estimated the individual loss aversion parameter *λ* to assess participants’ sensitivity to losses versus gains, calculated as *λ* = −β_loss_/β_gain_ (see Materials and methods, Experimental design, *Loss aversion task* for details). The mean *λ* value in our participants was 1.85, which was similar to previous research [7,26,66]. If loss aversion explained the reward-learning interference, we expected that individual λ values would correlate with the magnitude of enhanced exploration and/or reduced correct rates.

For self-reported incentive ratings, a 2 (motivation) × 3 (monetary amount: 1, 5, or 25 TWD) repeated-measures ANOVA showed no significant main effects, interactions, or post hoc comparisons (all *p* ≥ 0.054), indicating comparable motivation to gain rewards and avoid punishments across the three monetary amounts in Experiment 3. As in Experiments 1 and 2, RL_CON_ and PL_CON_ correct rates were significantly above chance (all *p* < 0.001; S1 Table), confirming successful learning.

**Fig 6:**
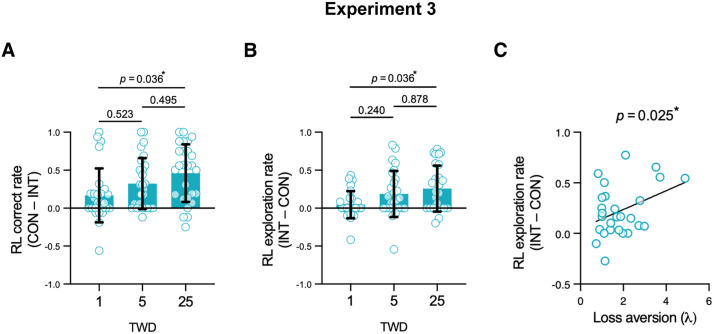
Monetary amount-dependent reward-learning interference, exploration, and its association with loss aversion in Experiment 3. **(A)** Reward-learning interference (reduced correct rate in reward-learning interference, RL_INT_, relative to reward-learning control, RL_CON_, trials) was greatest at 25 TWD, followed by 5 TWD and 1 TWD, with a significant difference between 1 and 25 TWD (*p* = 0.036). **(B)** Exploration rates in RL_INT_ vs. RL_CON_ trials followed the same trend, with a significant difference between 1 and 25 TWD (*p* = 0.036). **(C)** Increased exploration in RL_INT_ trials relative to RL_CON_ trials correlated with individual loss aversion (pooled across 5 and 25 TWD; *r* = 0.380, *p* = 0.025, one-tailed). All *p* values in (A) and (B) are Bonferroni-corrected in repeated-measures ANOVAs. Data are represented as mean ± SD. The data underlying Fig 6A and 6B can be found at https://doi.org/10.17605/OSF.IO/T7YWA.

By conducting 2 (learning type) × 2 (interference type) repeated-measures ANOVAs on correct rates, we found significant interactions for both the 5 TWD (*F*_1,26_ = 20.95, *p* < 0.001) and 25 TWD conditions (*F*_1,26_ = 25.67, *p* < 0.001), replicating the interference pattern observed in Experiments 1 and 2 (Fig 2B and 2C). The interaction for the 1 TWD condition was not significant (*p* = 0.063; S1 Table). Notably, reward-learning interference was greatest in the 25 TWD condition, moderate in the 5 TWD condition, and lowest in the 1 TWD condition (Fig 6A). A one-way repeated-measures ANOVA confirmed a main effect of monetary amount on the extent of reward-learning interference (*F*_2.0,50.6_ = 3.86, *p* = 0.028), with post hoc comparisons showing a significant difference between the 1 and 25 TWD conditions (*p* = 0.036). No such pattern of interference was observed in punishment learning (*p* = 0.548 for the main effect of monetary amount).

Consistent with Experiments 1 and 2, Model 6 provided the best fit to the behavioral data in Experiment 3 (S2 Table). Notably, in Experiment 3, directional posterior evidence for group-level mean differences in the interference parameter *S* was observed in the 5 and 25 TWD sessions, but not in the 1 TWD session, paralleling the behavioral interference effects observed across monetary amounts. Across Experiments 1–3, the mean condition difference in *S* was negative in 9 of 11 sessions, indicating an overall tendency for reduced *S* in RL_INT_ relative to RL_CON_. Consistent with Experiments 1 and 2, no session in Experiment 3 showed posterior probability >0.90 for condition differences in the natural logarithm of the inverse temperature parameter (*β*) or perseveration parameter (*ρ*; S7 Fig and S3 Table).

In both the 5 and 25 TWD conditions, exploration rates were significantly higher in PL_CON_ and RL_INT_ trials compared to RL_CON_ trials (RL_CON_ versus PL_CON_: both *p* < 0.001; RL_CON_ versus RL_INT_: both *p* ≤ 0.002, one-tailed; Fig 4E, S4 Table). The increase in exploration rate from RL_CON_ to RL_INT_ trials—averaged across the 5 and 25 TWD conditions, with the 1 TWD condition excluded because it did not show significant interference—positively correlated with reward-learning interference (*r* = 0.411, *p* = 0.017, one-tailed; Fig 4F). By contrast, changes in exploration rates between PL_INT_ and PL_CON_ trials did not significantly correlate with changes in correct rates (*p* = 0.100).

Importantly, mirroring the pattern in correct rates (Fig 6A), the increase in exploration in RL_INT_ relative to RL_CON_ trials in Experiment 3 also exhibited a graded increase with rising monetary amounts, with a significant effect of amount confirmed by a one-way repeated-measures ANOVA (*F*_1.9,49.5_ = 4.24, *p* = 0.021), driven primarily by the contrast between the 1 and 25 TWD conditions (*p* = 0.036, one-tailed, post hoc comparisons; Fig 6B). Furthermore, although individual *λ* values did not significantly correlate with reward-learning interference (*p* = 0.852), they significantly predicted the increase in exploration rates during RL_INT_ trials relative to RL_CON_ trials (*r* = 0.380, *p* = 0.025, one-tailed; Fig 6C).

Together, the monetary amount-dependent effects on performance and exploration, along with the linear relationship between individual *λ* and exploration rate, indicate that loss aversion is linked to enhanced exploration.

### Experiment 4: Dopamine system in reward-learning interference by punishment learning

Finally, given the dopaminergic system’s role in exploration, we tested whether reducing dopaminergic neurotransmission would alter the behavioral interference effect and attenuate the enhanced exploration. To this end, we conducted a between-subject study using a single dose of amisulpride (400 mg, a dopamine D2/3 receptor antagonist; *N* = 34) or placebo (lactose; *N* = 38). Experiment 4 was designed based on the pattern of effects observed across loss magnitudes in Experiment 3: task performance and exploration rates were comparable between the 1 and 5 TWD conditions and between the 5 and 25 TWD conditions, but differed significantly between the 1 and 25 TWD conditions (Fig 6A and 6B). Accordingly, to maximize sensitivity to loss magnitude while maintaining a feasible pharmacological design, Experiment 4 included two counterbalanced sessions using the 1 and 25 TWD loss amounts, which represent the lower and upper bounds of the behavioral effect observed in Experiment 3 (see Materials and methods, Experimental design, *Pharmacological manipulation*).

Upon arrival, each participant completed baseline physiological (blood pressure, heart rate), behavioral (affective, cognitive), and venous blood (prolactin) assessments. The learning task (identical to Experiment 1, with only monetary losses as punishment) began approximately 60 min after amisulpride administration, coinciding with its first peak blood concentration. Physiological, behavioral, and blood measures were reassessed immediately before and after the learning task.

For self-reported incentive ratings, a 2 (motivation) × 2 (monetary amount: 1 or 25 TWD) × 2 (drug: amisulpride or placebo) mixed ANOVA showed no significant three-way interaction (*p* = 0.881), suggesting that the effect of monetary amount on motivation to pursue rewards and avoid punishments was comparable between the amisulpride and placebo groups. These findings indicate that amisulpride did not influence motivation for rewards or punishments. Consistent with Experiments 1–3, correct rates in RL_CON_ and PL_CON_ trials were significantly above chance in both groups (all *p* < 0.001; S1 Table), indicating successful instrumental learning.

Baseline plasma prolactin levels were comparable between the placebo and amisulpride groups (*p* = 0.978). A 2 (drug) × 2 (time point: baseline or before the start of learning task) mixed ANOVA revealed a significant interaction (*F*_1,68_ = 118, *p* < 0.001) and a significantly elevated prolactin level in the amisulpride group (*p* < 0.001, post hoc comparisons), indicating reduced central dopaminergic activity [67]. The proportion of excluded participants, based on task performance across RL_CON_ and PL_CON_ conditions (see Materials and methods, Participants), did not differ significantly between the amisulpride (11/45) and placebo (13/51) groups (*p* > 0.999), and task performance in RL_CON_ and PL_CON_ trials was comparable between both groups (all *p* ≥ 0.246), together suggesting that amisulpride did not affect control learning conditions.

As in Experiments 1–3, Model 6 best accounted for the behavioral data in both the placebo and amisulpride groups in Experiment 4 (S2 Table). In the placebo group, task performance and exploration rates replicated the patterns observed in Experiments 1–3. A 2 (learning type) × 2 (interference type) repeated-measures ANOVA on correct rates revealed a significant interaction in the 25 TWD condition (*F*_1,37_ = 9.32, *p* = 0.004) but not in the 1 TWD condition (*p* = 0.696; S1 Table). In the 25 TWD condition, post hoc comparisons demonstrated that the correct rate in RL_INT_ trials was significantly lower than in RL_CON_ trials (*p* < 0.001), while the difference between PL_CON_ and PL_INT_ trials was not significance (*p* > 0.999). Regarding exploration in the 25 TWD condition, where reward learning was interfered with by punishment learning, RL_INT_ trials had a higher exploration rate than RL_CON_ trials (*p* = 0.001, one-tailed) and comparable exploration to PL_CON_ trials (*p* = 0.123, one-tailed; S4 Table).

**Fig 7:**
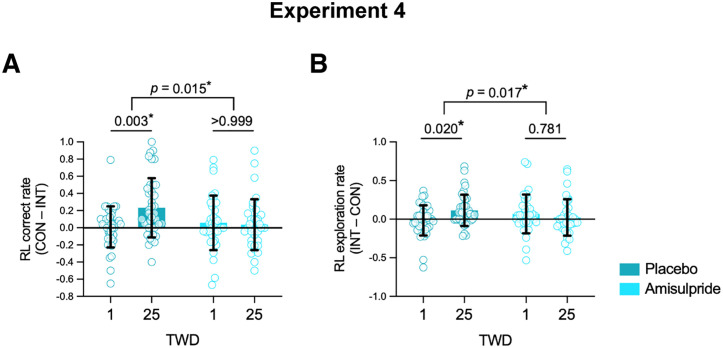
Effect of dopamine antagonist treatment on exploration and reward-learning interference in Experiment 4. **(A)** Treatment significantly reduced reward-learning interference (i.e., the decrease in correct rates in reward-learning interference, RL_INT_, relative to reward-learning control, RL_CON_, trials) for the 1 vs. 25 TWD condition (*p* = 0.015). The reduction in correct rates from RL_CON_ to RL_INT_ was larger at 25 TWD than at 1 TWD in the placebo group (post hoc comparisons: *p* = 0.003), with no difference in the amisulpride group (*p* > 0.999). **(B)** Treatment also significantly affected the difference in exploration rates between RL_INT_ and RL_CON_ trials across the 1 and 25 TWD conditions (*p* = 0.017). The increase in exploration rate from RL_CON_ to RL_INT_ was larger at 25 TWD than at 1 TWD in the placebo group (post hoc comparisons: *p* = 0.020), with no difference in the amisulpride group (*p* = 0.781). All *p* values correspond to interaction effects in mixed ANOVAs. Data are represented as mean ± SD. The data underlying this Figure can be found at https://doi.org/10.17605/OSF.IO/T7YWA.

Interestingly, in the amisulpride group, the learning-by-interference interaction was nonsignificant for both the 1 TWD (*p* = 0.125) and 25 TWD (*p* = 0.501) conditions (S1 Table). Post hoc comparisons showed that correct rates in RL_INT_ trials for the 25 TWD condition were no longer significantly different from those in RL_CON_ trials (*p* = 0.846). A 2 (drug) × 2 (monetary amount) mixed ANOVA on the correct rate difference between RL_CON_ and RL_INT_ trials showed a significant interaction (*F*_1,70_ = 6.25, *p* = 0.015; Fig 7A), indicating that amisulpride attenuated reward-learning interference by punishment learning more in the 25 TWD condition relative to the 1 TWD condition. Post hoc comparisons showed that the reduction in correct rates in RL_INT_ relative to RL_CON_ trials differed significantly between the 1 TWD and 25 TWD conditions in the placebo group (*p* = 0.003), but not in the amisulpride group (*p* > 0.999; Fig 7A). Similar to correct rates, while exploration rates in PL_CON_ trials remained significantly higher than in RL_CON_ trials (both *p* < 0.001, one-tailed), exploration rates in RL_INT_ trials became nonsignificant compared to RL_CON_ trials in the 25 TWD condition (*p* = 0.303, one-tailed; S4 Table). A 2 (drug) × 2 (monetary amount) mixed ANOVA on the exploration rate difference between RL_CON_ and RL_INT_ trials also revealed a significant interaction (*F*_1,70_ = 5.97, *p* = 0.017; Fig 7B), suggesting that amisulpride mitigated the exploration increase associated with reward-learning interference by punishment learning, particularly in the 25 TWD condition. Post hoc comparisons showed that the increased exploration rate in RL_INT_ relative to RL_CON_ trials differed significantly between the 1 TWD and 25 TWD conditions in the placebo group (*p* = 0.020), but not in the amisulpride group (*p* = 0.781; Fig 7B). These behavioral effects occurred without differences in heart rate, blood pressure, mood, or working memory performance across the three time points (baseline, before the learning task, and after the learning task) between the amisulpride and placebo groups (all *p* ≥ 0.261). Individual *λ* values did not significantly differ between the two groups (*p* = 0.461).

## Discussion

Across four experiments, we found that reward learning performance declined when choices had both reward and punishment histories, compared to when only reward learning was involved. The extent of this decline covaried with enhanced exploratory behavior elicited by a reward learning choice’s punishment learning history and was further modulated by the magnitude of potential losses. This enhanced exploration entailed neural activity in the dmPFC, was associated with individual loss aversion behavior, and was abolished following dopamine antagonist treatment. Collectively, these findings support our hypothesis that reward-learning interference by punishment learning is associated with loss aversion-related increases in exploration and is modulated by dopaminergic mechanisms.

### Impact of prior punishment history on reward-learning interference

Despite extensive research on the neural correlates of reward and punishment learning, most human studies have investigated these processes separately, using trial structures that isolate either reward or punishment learning [3–8]. Other paradigms have employed bivalent cues that signal both rewarding and punishing outcomes simultaneously within a single trial [9–11], thereby capturing one form of conflict when a single choice leads to mixed outcomes. Our approach complements this line of work by probing a different type of conflict—namely, how learning is influenced by prior, context-dependent reinforcement histories. Specifically, we used overlapping *AB* and *BC* stimulus pairs, in which the same option was associated with reward learning in one context and punishment learning in another. This design required participants to integrate or prioritize conflicting value signals acquired across trials and contexts, thereby engaging a more dynamic form of value conflict than that elicited by simultaneous outcomes within a single trial. By examining how the brain resolves this type of interference, our findings contribute to a broader understanding of how reward and punishment learning interact in reinforcement learning.

Across Experiments 1 and 2, we consistently observed impaired reward learning when choices were also associated with punishment learning experiences, regardless of whether involving primary (pain) or secondary (monetary loss) punishment. In contrast, punishment learning was unaffected by the reward learning history of the choices. This reward-learning interference phenomenon cannot be attributed to incentive-related confounds, contextual influences, or differences in associability or uncertainty, as the *B* option in both reward (*AB* pair) and punishment (*BC* pair) learning trials in Experiment 1 had identical probabilities (25.0%) of yielding rewarding or punishing outcomes [18]. Model-based analyses supported this finding, as model comparisons indicated that the model including the interference term at the choice phase significantly outperformed alternatives after controlling for perseveration.

Taken together, these results suggest that the interference of reward learning by punishment learning arises from a valence-dependent asymmetry in processing positive and negative information. Prior punishment history leads individuals to prioritize avoiding punishment over seeking rewards. This differs from the positivity bias observed in other learning processes [68], such as attentional focus on reward-predictive cues [18–20] and the updating of instrumental values [10,69,70].

Consistent with this interpretation, several findings support the functional relevance of the interference parameter *S* despite variability across experiments and sessions. Reduced *S* in RL_INT_ relative to RL_CON_ emerged in a subset of sessions, particularly in the 5 and 25 TWD sessions of Experiment 3, which also showed robust behavioral interference effects. Moreover, the mean $\Delta S_{{RL}_{INT}}$ was negative in nearly all sessions, consistent with the hypothesis that punishment-learning history influences reward-learning choices. By contrast, there was no evidence for condition-dependent modulation of the inverse temperature or perseveration parameters. Posterior predictive simulations further showed that inclusion of *S* improved model fit and reproduced the reduced reward-learning performance under interference. Together, these findings suggest that reward-learning interference is better captured by punishment-history-dependent interference at choice. Notably, Experiment 3, which involved only monetary loss as punishment, showed the clearest condition-dependent modulation of *S* relative to Experiments 1 and 2, which included both monetary loss and pain, suggesting that punishment modality or salience may contribute to heterogeneity in parameter estimates. Future work should clarify how different punishment contexts shape interactions between reward and punishment learning.

### Enhanced exploration associated with reward-learning interference by punishment learning

Across all four experiments, the punishment-learning control condition (PL_CON_ trials) consistently showed higher exploration rates than the reward-learning control condition (RL_CON_ trials). This is consistent with prior research demonstrating that negative outcomes tend to entail greater exploration relative to positive outcomes [29,35–37]. Despite this difference in exploration rates, participants learned successfully in the two control learning conditions, supporting the notion that successful reward learning requires a more exploitative approach [29,35], whereas successful punishment learning engages a more exploratory strategy. Notably, we showed that when reward learning choices involved punishment histories characterized by higher and unequal (i.e., two punishment options with different probabilities) punishment rates, participants exhibited higher exploration rates than when punishment histories were relatively lower and equal (i.e., two punishment options with identical probabilities). The higher the increase in exploration rates, the worse the task performance in reward learning. Together with the notion that exploration engages cognitive control mechanisms that suppress exploitation and switch to exploratory strategies [28,38,62], we propose that the punishment learning history of a choice in reward learning may be associated with a disruption of the exploration-exploitation balance, contributing to impaired reward-learning performance.

To strengthen the link between enhanced exploration and observed reward-learning interference at the neural level, we analyzed our fMRI data, focusing on brain regions involved in exploration. Our identification of the dmPFC in exploration is consistent with previous research [31,63,71]. Evidence suggests that the dmPFC processes feedback to balance exploration and exploitation [72] and to evaluate alternatives relative to the current option to guide exploration [73]. More importantly, the positive association between increased activation in the dmPFC and reward-learning interference by punishment learning provides neural evidence consistent with the behavioral link between enhanced exploration and interference.

Two issues regarding exploration merit further discussion. First, exploration and task performance are defined in distinct reference frames in our design. Exploration is indexed by choices deviating from model-derived subjective expected values, whereas correct rates reflect the selection of objectively superior options. Consequently, these two metrics are conceptually dissociable. Consistent with this distinction, correct rates remained comparable between RL_CON_ and PL_CON_ trials despite significantly higher exploration in the latter, demonstrating that exploratory choices are not inherently erroneous. Increased exploration did not systematically predict reduced accuracy in PL_INT_ relative to PL_CON_ trials, suggesting that variation in accuracy cannot be trivially explained by exploration rates. Moreover, the neural dissociation—where exploration-related dmPFC activity tracked reduced correct rates in RL_INT_ relative to RL_CON_ trials but not in PL_INT_ relative to PL_CON_ trials—further indicates that exploration cannot be equated with task errors. In conjunction with matched objective outcome probabilities between reward- and punishment-learning pairs, we posit that the selective impairment of reward learning and the accompanying increase in exploration following punishment learning cannot be attributed to task structure, but instead reflect altered learning processes induced by prior punishment experience.

Second, in the present study, exploration was defined as a model-informed behavioral index, following previous work [29,35–37,39,40], rather than as a direct estimate of a specific computational parameter. The parameter *β* did not differ across conditions, indicating that the observed reward-learning interference is not driven by increased choice stochasticity. Instead, the behavioral effects are better explained by systematic changes in decision processes, such as interference captured by parameter *S*. Unlike restless-bandit tasks that explicitly model uncertainty to capture directed exploration [31,38,74,75], our task employed static outcome probabilities [30], and therefore did not include an uncertainty-bonus mechanism. Whether other forms of exploration, such as directed exploration or alternative computational mechanisms, contribute to the observed interference remains an open question for future research.

### Loss aversion-dependent increase in exploration

While the monetary amount-dependent nature of reward-learning interference supports a role for loss aversion, the absence of a significant relationship between this interference and individual loss aversion behavior suggests that other mechanisms may mediate the link between loss aversion and reward-learning interference. Since reward-learning interference was associated both with prior punishment history and with enhanced exploration, as discussed above, we investigated whether individual loss aversion—a negativity bias seen in gain-loss contexts [22]—was directly related to exploration. We found that, like reward-learning interference, the exploration rate exhibited a monetary amount-dependent pattern across the three monetary levels in Experiment 3, consistent with the magnitude-dependent nature of loss aversion [64,65]. Furthermore, individual loss aversion behavior, represented by the parameter *λ* assessed separately, parametrically predicted the increased exploration rate when a reward learning choice included punishment learning histories, compared to when it did not. This positive correlation, alongside the linear relationship between enhanced exploration and reward-learning interference, provides an indirect pathway connecting individual loss aversion to reward-learning interference. We propose that the observed reward-learning interference may stem from individual loss aversion through its relationship with exploratory behavior. These findings highlight the overlooked link between loss aversion and reinforcement learning [7,76] while offering deeper insights into its neural basis.

### The role of dopamine in reward-learning interference by punishment learning

Lastly, using amisulpride to attenuate dopaminergic neurotransmission, we found that the interference with reward learning was eliminated, accompanied by the abolishment of increased exploration. This reduction in exploration is consistent with the established role of dopamine in regulating exploratory behavior [32,33,77]. Despite dopamine’s well-documented involvement in reward learning [6,78], performance in the control reward learning condition, as well as individual loss aversion, remained unaffected at the administered dose of amisulpride. These findings suggest that, in the absence of changes to loss aversion or isolated reward and punishment learning, dopaminergic attenuation links heightened exploration with impaired reward learning, supporting a role for dopaminergic neurotransmission in punishment-history-related reward maximization failure and associated increases in exploration. Furthermore, our findings emphasize the central role of dopaminergic signaling in regulating learning when reward learning occurs within punishment contexts, necessitating adaptive control of exploratory behavior. Although our study focuses on healthy individuals, the results contribute to a broader framework of dopamine’s role in reinforcement learning and may inform understanding of neuropsychiatric conditions characterized by dopaminergic dysregulation and exploration–exploitation deficits, such as schizophrenia [34], substance addictions [79], gambling disorder [42], and Parkinson’s disease [80]. Given the between-subject pharmacological design of Experiment 4, future work using within-subject approaches may further refine our understanding of dopaminergic contributions to reward-learning interference.

In conclusion, while performing a reward learning task where the reinforcement history of a choice relates to punishment learning, human behavior reflects a reward maximization failure, prioritizing punishment avoidance over reward acquisition. This punishment-history interference during reward learning is associated with increased exploration and depends on dopaminergic mechanisms, with individual differences in exploration linked to loss aversion. These results offer a mechanistic account of how the brain resolves competing value signals and deepen our understanding of dopamine’s role in reinforcement learning.

## Materials and methods

### Ethics statement

All participants provided written informed consent and received compensation for their participation. The study was approved by the Ethics Committee of National Taiwan University Hospital, Taipei, Taiwan (approval numbers: 201812184RINB and 202402044MINE), and conducted in accordance with the Declaration of Helsinki.

### Participants

All participants were healthy, right-handed adults with no history of neuropsychiatric disorders. In Experiment 1, 5 participants were excluded for correct rates below chance (50%) across RL_CON_ and PL_CON_ conditions, and 6 for excessive head motion during fMRI scanning. The final analysis included 30 participants (15 females; aged 20–34 years; mean ± SD = 24.1 ± 3.9 years). In Experiment 2, 8 participants were excluded for below-chance performance, and 5 for excessive head motion, resulting in a final sample of 30 participants (13 females; age: 20–31; mean ± SD = 22.4 ± 2.5 years). In Experiment 3, 15 were excluded for below-chance performance at any of the three monetary levels, leaving 27 participants (16 females; age range: 20–29; mean ± SD = 23.3 ± 2.6 years) for final analysis. To determine the appropriate sample size for the interference effect, a pilot study (*N* = 14; 8 females; age range: 20–25 years, mean ± SD = 21.6 ± 1.8 years) revealed an effect size of *d* = −0.56. A power analysis performed using G*Power [81] indicated that a sample size of 27 would achieve 80% power to detect this effect size at an *α* level of 0.05 (two-tailed). In Experiment 4, 24 participants (11 in amisulpride group and 13 in control group) were excluded for below-chance performance at any monetary level, resulting in 72 male participants (34 in the amisulpride group: aged 19–28 years, mean ± SD = 22.2 ± 2.8 years; 38 in the placebo group: aged 18–28 years, mean ± SD = 22.8 ± 2.7 years). To avoid confounding effects of menstrual cycle-related hormonal fluctuations on dopaminergic function [82,83], we restricted recruitment to male participants.

### Stimuli

#### Visual stimulation.

Prior to the formal experiment, a pretest with 7 independent participants selected 36 oracle bone script characters, which were divided into 4 sessions (9 characters per session) for the formal learning task (see below for details). Stimuli were matched for moderate visual complexity, low familiarity, and low verbalizability to control for perceptual, memory, and language confounds [84,85]. To further mitigate potential confounds in memory and language processing during the formal experiment, each character was presented in inverted orientation [86].

#### Electrocutaneous stimulus.

Painful electrocutaneous stimuli were delivered using a bipolar constant-current stimulator (DS5, Digitimer) to the dorsum of the participant’s left hand via MRI-compatible leads (LEAD108, Biopac Systems) and silver chloride surface electrodes (EL-508, Biopac Systems). Each stimulus consisted of four monophasic rectangular pulses (pulse width: 2 ms at 20 Hz). Stimulus delivery and behavioral data acquisition were synchronized using Presentation (Neurobehavioral Systems) and LabVIEW software (National Instruments).

### Experimental design

#### Incentive calibration (Experiments 1–4).

To examine how valences interact in reinforcement learning, we calibrated reward and punishment magnitudes for each participant before each experiment, equating punishments (painful stimulation or monetary loss) to the reward magnitude used in that session: 5 TWD in Experiments 1 and 2, 1, 5, or 25 TWD in Experiment 3, and 1 or 25 TWD in Experiment 4. This procedure ensured comparable subjective incentive values and task performance [8] between pursuing rewards and avoiding punishments, given the potential bias of incentive salience on instrumental behavior [87]. For the calibration of monetary gain versus painful stimulation, participants were exposed to individually calibrated painful stimuli corresponding to a pain score of 50/100 (i.e., moderate pain) [88], and then chose between receiving 5 TWD and avoiding the pain. The stimulus intensity was adjusted until subjective equivalence was reached (mean ± SD: 3.5 ± 4.4 mA in Experiment 1 and 4.0 ± 3.6 mA in Experiment 2 in our participants). For monetary gain versus monetary loss, participants chose between obtaining 5 TWD and avoiding a monetary loss, with the loss amount adjusted to achieve equivalence (mean ± SD: −5.7 ± 1.5 TWD in Experiment 1; −8.6 ± 10.2 TWD in Experiment 2; −1.4 ± 0.7, −5.3 ± 2.2, and −23.8 ± 10.8 TWD for rewards of 1, 5, and 25 TWD, respectively, in Experiment 3). The calibrated pain intensity and monetary loss amounts were used in the formal learning task for the participant.

#### Learning task (Experiments 1–4).

Experiment 1 comprised four sessions of learning task across two visits, separated by one week. On one visit, participants completed two learning sessions with monetary gain as the reward and painful stimulation as the punishment (pain-related learning task). On the other, they completed two sessions with monetary gain as the reward and monetary loss as the punishment (monetary loss-related learning task). The order of the tasks was counterbalanced across participants. Each session introduced 9 unique oracle bone script characters, grouped into 6 pairs, each repeated 16 times, with pair presentations randomized. These pairs were further organized into 3 clusters of interrelated pairs, where overlapping options connected adjacent pairs within each cluster (i.e., *AB* linked to *BC*, *DE* linked to *EF*, and *GH* linked to *HI*; Fig 1B). For reward learning pairs *AB* and *DE*, selecting the best option (*A* and *D*) resulted in a 68.8% chance of receiving a 5 TWD reward (11 out of 16 trials) and a 31.2% chance of receiving nothing (no reward or punishment; 5 out of 16 trials), while the worse option (*B* or *E*) offered a 25.0% chance of reward (4 out of 16 trials) and a 75.0% chance of nothing (12 out of 16 trials). For punishment learning pairs *BC* and *HI*, the better option (*B* or *H*) carried a 25.0% chance of punishment and a 75.0% chance of nothing, whereas the worse option (*C* or *I*) had a 68.8% chance of punishment and a 31.2% chance of nothing. The *EF* and *GH* pairs had equal outcome probabilities across options, so no option was objectively better than the other within these trials [89], though participants might still exhibit punishment avoidance. Compared to *EF* (each option 25.0% punishment), the *BC* pair had a higher overall punishment rate (*B*: 25.0%; C: 68.8%), which was expected to elicit stronger loss-avoidant behavior relative to *EF*, and thus influence reward learning in *AB* relative to *DE*. Similarly, compared to *GH*, the *AB* pair had a higher overall reward rate and asymmetrical outcome probabilities, expected to enhance reward-seeking tendencies relative to *GH*, and thus influence punishment learning in *BC* relative to *HI*. Therefore, this design enabled examination of whether punishment avoidance modulated the effect of punishment history on reward learning (*AB* versus *DE*) and whether reward preference modulated the effect of reward history on punishment learning (*BC* versus *HI*). In each trial, participants chose between the left or right option by pressing one of the corresponding buttons with their right index or middle finger within a 2.5 s time window. After selection, the chosen option remained on screen for 1 s, followed by a 2 s fixation period, a 2 s outcome presentation, and a 2–4 s jittered intertrial interval (Fig 1A).

Participants were told to optimize performance by maximizing gains and avoiding losses, without knowledge of option-outcome contingencies. They were informed that their payoff would be performance-based, with an average payoff of 1,000 TWD. A practice session, which was a shortened version of the task (4 repetitions of each pair), was conducted prior to the formal task to familiarize participants with the procedure. After each session, participants provided incentive ratings on a visual analogue scale (0: not at all; 100: very much), indicating their motivation to pursue rewards (e.g., “How motivated are you to win 5 TWD?”) and to avoid punishments (e.g., “How motivated are you to avoid losing 5 TWD (or experiencing this painful stimulation)?”).

Experiment 2 followed the same procedure as Experiment 1, except that the probability composition of options *A* and *B* in the *AB* pair was reversed (Fig 1C). Since Experiments 1 and 2 showed similar interference of punishment learning with reward learning across punishment types (monetary loss and pain) and regardless of whether *A* and *B* had probabilities of 68.8% and 25.0% or were reversed, Experiments 3 and 4, which did not involve scanning, focused solely on monetary loss-related learning tasks and required a single visit. Experiment 3 followed the probability composition of Experiment 2 and included three counterbalanced sessions with loss amounts of 1, 5, or 25 TWD. Participants also completed a separate loss aversion task (see below), with the order of learning and loss aversion tasks counterbalanced. Experiment 4, which basically followed the probability composition of Experiment 1, included two counterbalanced sessions with loss amounts of 1 or 25 TWD and incorporated pharmacological manipulation (see below).

#### Loss aversion task (Experiment 3).

Experiment 3 consisted of the learning task described above and a loss aversion task assessing each participant’s loss aversion [26], with task order counterbalanced. This loss aversion task consisted of 144 randomly presented trials. On each trial, participants were asked to accept or reject a mixed gamble with a 50% chance of either a monetary gain (ranging from 200 to 640 TWD in increments of 40 TWD, resulting in 12 values) or a monetary loss (ranging from 100 to 320 TWD in increments of 20 TWD, resulting in 12 values). Participants were informed that at the end of the experiment, one trial would be randomly selected, and the outcome of that trial would affect their final payoff. We used logistic regression (function *fitglm* in Matlab) to model the probability of accepting the gamble, with the magnitude of potential gain and loss as independent variables. Individual loss aversion was defined as *λ* = −*β*_loss_/*β*_gain_ [7,26], which captures the relative weighting of losses versus gains in decision-making, with higher values indicating a stronger behavioral tendency to avoid losses over acquiring equivalent gains.

#### Pharmacological manipulation (Experiment 4).

In Experiment 4, we employed a randomized, double-blind, between-subject design to investigate whether the dopaminergic system mediates the interference of reward learning by punishment learning through its effects on exploratory behavior. Participants received a single oral dose of either 400 mg amisulpride (a dopamine antagonist; *N* = 34), 150 mg L-Dopa (a dopamine precursor), or a placebo (lactose; *N* = 38). Amisulpride was selected for its selective blockade of D2/D3 receptors, reducing dopaminergic neurotransmission without affecting non-dopaminergic receptors [90]. This dose has been reported to be well tolerated without significant side effects in healthy individuals [91]. Before participation, all participants were informed of the general clinical use and potential adverse effects of the administered drugs. No participant reported any side effects.

Participants arrived at the laboratory approximately 60 min before the start of the learning task and completed baseline physiological and behavioral assessments, including heart rate, blood pressure, mood state (measured using the short-form Profile of Mood States) [92], and working memory capacity (assessed via digit span). A venous blood sample was collected to determine baseline prolactin levels. Participants then received their first pill (amisulpride or placebo), followed by a second pill 30 min later (L-Dopa or placebo). The amisulpride, L-Dopa, and placebo groups received amisulpride/lactose, lactose/L-Dopa, and lactose/lactose, respectively. To maintain blinding, all drug conditions were administered in identical gelatin capsules. Thirty minutes after taking the second pill, participants began the learning task (described above). Drug administration timing was based on pharmacokinetic data indicating that the first peak of amisulpride occurs at around 60 min [90], while L-Dopa reaches peak plasma concentration approximately 30 min post-administration.

Immediately before and after the learning task, participants underwent the same physiological and behavioral assessments as well as blood sampling, as conducted at baseline. Behavioral data were analyzed to examine whether dopamine-induced changes influenced cognitive or affective measures, while blood samples were used to assess changes in prolactin, an inverse marker of central dopaminergic activity [67]. Given that the primary objective here was to examine the effects of reducing dopaminergic neurotransmission on the interference of reward learning by punishment learning, the analysis focused on the amisulpride and placebo groups, where dopamine blockade could be directly assessed. Findings from the L-Dopa group will be reported elsewhere.

### Behavioral analysis and computational modeling of the learning task

In the present study, individual task performance was measured by the correct rate (i.e., the percentage of selecting the best option) in the four asymmetric pairs (i.e., *AB*, *BC*, *DE*, and *HI* pairs). The extent of reward-learning interference by punishment learning was calculated as the difference in correct rates between RL_CON_ and RL_INT_ trials, and the extent of punishment-learning interference by reward learning was quantified as the difference in correct rates between PL_CON_ and PL_INT_ trials.

#### Model specification and fitting.

Guided by our hypothesis that reward-learning interference arises from punishment learning in the interrelated pair, and informed by the model-neutral findings from Experiments 1 and 2—which provided no evidence for contextual effects—our computational modeling analyses focused primarily on three models: Perseveration, Perseveration + Interference at Choice, and Perseveration + Context. These models allow us to test the interference mechanism while appropriately controlling for perseveration—the tendency to repeat previous choices regardless of expected value or uncertainty [31,50–52]—and, consistent with the empirical pattern observed in Experiments 1 and 2, ruling out contextual influences. To ensure that our conclusions did not depend on this theoretically motivated focus, we also evaluated 16 additional models representing all other combinations of the three key components (perseveration, interference, and context). Model comparisons across all 19 models are reported in S2 Table.

Each participant’s choices were modeled using these computational models, each combining a specific learning rule with a choice rule. The learning rule was based on the delta rule, which updates expected option values trial by trial [46,93] and has been widely employed in reinforcement learning research [5,6,8,10]. We allowed separate learning rates for each reward- and punishment-learning pair, and included state-specific Q-learning architectures in which the value of an option was allowed to differ across the pairs in which it appeared (e.g., the value of option *B* could differ between the *AB* and *BC* pairs). These additional learning rules capture conditional or occasion-setting mechanisms used in prior reinforcement-learning studies [5,94] and were incorporated into the full set of 19 models. The learning rule could include or omit choice context value [5,53]. The choice rule was based on a softmax function, which is standard in models of reinforcement learning and exploration [31,38,95]. To account for potential confounds due to perseveration, the choice rule could include or omit a perseveration term. Critically, we included a free parameter *S* to model individual sensitivity to the outcomes from the interrelated pair. This parameter scaled with prior exposure to oppositely valenced outcomes (*L*), constituting an interference term that could modulate either the learning phase (via the learning rate or prediction error) or the choice phase. Because the precise computational stage at which interference occurs is unknown, this term was implemented to capture potential effects at either stage (S2 Table).

Given that pain magnitudes in Experiments 1 and 2 were individually calibrated to be psychologically equivalent to the corresponding reward magnitude (5 TWD), outcomes were coded according to the valence-based coding scheme, with reward coded as +1 and pain coded as −1. The structure and rationale of selected example models are described below.

***Model 1: Basic:*** In the basic model (i.e., no context value, no perseveration, and no interference term), the expected value of the chosen option was updated as follows:


$$\begin{array}{c} Q_{\left(t+\text{1}\right)}=Q_{\left(t\right)}+\alpha \times \delta_{\left(t\right)} \end{array}$$

$$\begin{array}{c} \delta_{\left(t\right)}=\;R_{\left(t\right)}-Q_{\left(t\right)} \end{array}$$

where *Q*_(*t+1*)_ is the expected value of the chosen option on trial *t* + 1, updated from *Q*_(*t*)_ using the prediction error *δ*_(*t*)_ multiplied by the learning rate *α*. The overlapping options (*B*, *E*, and *H*) were assigned separate *Q*-values across different pairs. All *Q*-values were initialized to 0 at the start of the task and updated only when the corresponding pair was encountered. The prediction error *δ*_(*t*)_ reflects the discrepancy between the actual outcome *R*_(*t*)_ and the expected value *Q*_(*t*)_ at trial *t*.

The choice rule used a standard softmax:


$$\begin{array}{c} P_{i(t)}=\frac{\text{exp}(\beta Q_{i(t)})}{\sum_{j}\text{exp}(\beta Q_{j(t)})} \end{array}$$

where *P*_*i*(*t*)_ is the probability of choosing option *i* on trial *t*, *j* is the number of option in each pair, *β* is an inverse temperature parameter capturing choice stochasticity [96], and *Q*_*i*(*t*)_ is the expected value of option *i* on trial *t*.

Based on Model 1, we constructed two extensions to test whether value representations generalize across pairs (Model 1a) and whether asymmetric learning from positive and negative outcomes better explains behavior (Model 1b).

***Model 1a: cross-pair Q-value:*** In this model, the learning rule and choice rule were identical to Model 1, except that overlapping options shared a common value across pairs. Specifically, for overlapping options (e.g., *B* in *AB* and *BC*), a single *Q*-value was maintained and updated irrespective of pair identity:


$$\begin{array}{c} \overset{\text{shared}}{\underset{\left(t+\text{1}\right)}{Q}}=\overset{\text{shared}}{\underset{\left(t\right)}{Q}}+{\alpha \times \delta }_{\left(t\right)} \end{array}$$

$$\begin{array}{c} \delta_{\left(t\right)}=R_{\left(t\right)}-\overset{\text{shared}}{\underset{\left(t\right)}{Q}} \end{array}$$

where $\overset{\text{shared}}{\underset{\left(t\right)}{Q}}$ denotes the common value associated with the overlapping option across all pairs in which it appears. Thus, updates to an overlapping option propagate across pairs, enabling carry-over effects at the value level.

***Model 1b: Asymmetric learning rate:*** In this model, the choice rule was identical to Model 1, but separate learning rates were used for positive and negative prediction errors:


$$\begin{array}{c} Q_{t+\text{1}}=Q_{t}+\left\{ \begin{array}{cc} \alpha^{+} & \;\text{if }\delta_{t}>\text{0}\\ \alpha^{-} & \;\text{if }\delta_{t}<\text{0} \end{array}\right. \end{array}$$

$$\begin{array}{c} \delta_{\left(t\right)}=\;R_{\left(t\right)}-Q_{\left(t\right)} \end{array}$$

where *α*^+^ governs updating following better-than-expected outcomes (positive prediction errors), and *α*^−^ governs updating following worse-than-expected outcomes (negative prediction errors).

***Model 2: Interference at choice:*** This variant retained the learning rule from Model 1 but included the interference term in the choice rule:


$$\begin{array}{c} P_{i(t)}=\frac{\text{exp}(\beta (Q_{i(t)}\;+\;\gamma SL_{\left(t\right)}))}{\sum_{j}\text{exp}(\beta (Q_{j(t)}\;+\;\gamma SL_{\left(t\right)}))} \end{array}$$

where *γ* is an indicator function (1 if the chosen option was the overlapping option, i.e., options *B*, *E*, and *H*; 0 otherwise); *S* denotes the interference bonus parameter, which quantifies the degree to which reward learning was affected by punishment learning; and *L*_(*t*)_ reflects the ratio of prior experience (up to trial *t*) of oppositely valence outcomes after choosing the overlapping option to the total number of encounters with the interrelated pair.

***Models 3: Interference at learning rate:*** Here, the choice rule was the same as Model 1, but the interference term modulated the learning rate:


$$\begin{array}{c} Q_{\left(t+\text{1}\right)}=Q_{\left(t\right)}+\left(\alpha +\gamma SL_{\left(t\right)}\right){\times \delta }_{\left(t\right)} \end{array}$$

$$\begin{array}{c} \delta_{\left(t\right)}=\;R_{\left(t\right)}-Q_{\left(t\right)} \end{array}$$

***Model 4: Interference at prediction error:*** In this variant, the interference term affected the prediction error:


$$\begin{array}{c} Q_{\left(t+\text{1}\right)}=Q_{\left(t\right)}+\alpha \times \left(\delta_{\left(t\right)}+\gamma SL_{\left(t\right)}\right) \end{array}$$

$$\begin{array}{c} \delta_{\left(t\right)}=\;R_{\left(t\right)}-Q_{\left(t\right)} \end{array}$$

***Model 5: Perseveration:*** This variant added the perseveration term to the choice rule:


$$\begin{array}{c} P_{i(t)}=\frac{\text{exp}(\beta (Q_{i(t)}\;+\;I\rho ))}{\sum_{j}\text{exp}(\beta (Q_{j(t)}\;+\;I\rho ))} \end{array}$$

where *I* is an indicator function (1 if the chosen option was the same option chosen in the previous trial; 0 otherwise) and *ρ* is the perseveration bonus parameter.

Model 5 tests whether behavior is influenced by simple choice repetition from the preceding trial. To further characterize the temporal extent and generalization of perseveration, we constructed three extensions (Models 5a–5c): Model 5a allows perseveration to depend on multiple prior trials, Model 5b allows it to generalize across stimulus pairs, and Model 5c combines both extensions.

***Model 5a: Perseveration (trial-history):*** In this model, the choice rule extends Model 5 by allowing the perseveration term to incorporate information from multiple preceding trials rather than only the immediately preceding trial.


$$\begin{array}{c} P_{i(t)}=\frac{\text{exp}(\beta (Q_{i(t)}\;+\;H_{i(t)}))}{\sum_{j}\text{exp}(\beta (Q_{j(t)}\;+\;H_{j(t)}))} \end{array}$$

$$\begin{array}{c} H_{i(t)}=\sum_{k=\text{1}}^{K}w_{k}\cdot I_{t-k(i)} \end{array}$$

where *H*_*i(t)*_ denotes the perseveration term for option *i* at trial *t*, defined as a weighted sum of past choices across a history window: *H*_*i(t)*_ = sum from *k* = 1 to *K* of *w*_*k*_ times *I*_*t-k(i)*_. Here, *K* denotes the number of previous trials included in the history window, and *k* indexes the temporal lag from 1 to *K*. *I*_*t-k(i)*_ is an indicator function that equals 1 if option *i* was chosen at trial *t*-*k*, and 0 otherwise, and *w*_*k*_ denotes the weight associated with lag *k*, reflecting the influence of past choices across different time lags.

***Model 5b: Perseveration (cross-pair):*** In this model, the choice rule is again identical to Model 5, but the indicator *I* is defined across overlapping pairs. Specifically, *I* = 1 if the chosen option matches the option selected in the previous trial involving the same stimulus (e.g., choosing *B* in an *AB* trial following a previous choice of *B* in either an *AB* or *BC* trial), and *I* = 0 otherwise. This formulation directly tests for carry-over effects of perseveration across pairs.

***Model 5c: Perseveration (trial-history + cross-pair):*** This model combines the two extensions above by incorporating both multi-trial history and cross-pair generalization. Specifically, *H*_*i(t)*_ is defined as in Model 5a, while the indicator function *I* is defined over all trials involving the same stimulus, regardless of pair context (as in Model 5b). Thus, the perseveration term captures both temporal accumulation of past choices and carry-over effects across stimulus pairs.

***Model 6: Perseveration + interference at choice:*** In this variant, the learning rule matched the basic form, while the choice rule incorporated both the interference and perseveration terms:


$$\begin{array}{c} P_{i(t)}=\frac{\text{exp}(\beta (Q_{i(t)}\;+\;\gamma SL_{\left(t\right)}\;+\;I\rho ))}{\sum_{j}\text{exp}(\beta {(Q}_{j(t)}\;+\;\gamma SL_{\left(t\right)}\;+\;I\rho ))} \end{array}$$

Our model comparison identified Model 6 as the best-fitting model (see Results). Its estimated parameters were therefore used to derive trial-wise expected values and prediction error signals for each option, which served as regressors for the subsequent behavioral and fMRI analyses.

In the original parameterization of this model, condition differences are inferred from separately estimated condition-specific parameters, which can lead to greater uncertainty in between-condition comparisons. To provide a more interpretable parameterization for evaluating condition differences in model parameters, we therefore implemented a baseline-referenced reparameterization (baseline-referenced Model 6), in which the interference parameter in the RL_CON_ condition serves as the reference baseline and condition effects are expressed as deviations from this baseline:


$$\begin{array}{c} {\text{S}}_{\text{c}}=\left\{ \;\;\;\;\;\;\;\;\begin{array}{ccc} & S_{RL_{CON}} & \;\text{if }c={\text{RL}}_{\text{CON}}\\ & S_{RL_{CON}}+\Delta S_{{RL}_{INT}} & \text{ if }c={\text{RL}}_{\text{INT}}\\ & S_{RL_{CON}}+\Delta S_{{PL}_{CON}} & \;\text{if }c={\text{PL}}_{\text{CON}}\\ & S_{RL_{CON}}+\Delta {\text{S}}_{{PL}_{INT}} & \text{ if }c={\text{PL}}_{\text{INT}} \end{array}\right. \end{array}$$

Here, $S_{RL_{CON}}$ denotes the baseline reference parameter, and each ΔS term represents the deviation from this baseline for the corresponding condition. This parameterization introduces a shared baseline and enables direct estimation of condition differences, particularly for the primary contrast between RL_CON_ and RL_INT_.

***Model 6a: Perseveration (trial-history) + interference at choice:*** This model is identical to Model 6, except that the perseveration term incorporates multi-trial history as in Model 5a.

***Model 6b: Perseveration (cross-pair) + interference at choice:*** This model is identical to Model 6, except that perseveration generalizes across stimulus pairs as in Model 5b.

***Model 9: Context:*** When incorporating choice context value, the prediction error for each option was adjusted by the context value (*CV*) of the pair, serving as a reference point for outcome evaluation [5,53]:


$$\begin{array}{c} Q_{\left(t+\text{1}\right)}=Q_{\left(t\right)}+\alpha \times \delta_{\left(t\right)} \end{array}$$

$$\begin{array}{c} \delta_{\left(t\right)}=\;R_{\left(t\right)}-{CV}_{\left(t\right)}-Q_{\left(t\right)} \end{array}$$

This context value was itself updated by its own learning rate and prediction error:


$$\begin{array}{c} {CV}_{\left(t+\text{1}\right)}={CV}_{\left(t\right)}+\alpha_{CV}\times \delta_{CV(t)} \end{array}$$

$$\begin{array}{c} \delta_{CV(t)}=\;R_{CV(t)}-{CV}_{\left(t\right)} \end{array}$$

where *CV*_(*t+1*)_ is the expected context value at trial *t* + 1, updated from *CV*_(*t*)_ using its prediction error *δ*_*CV(t)*_ scaled by the context learning rate *α*_*CV*_. The prediction error *δ*_*CV(t)*_ reflects the discrepancy between the actual context outcome *R*_*CV(t)*_ and the expected context value *CV*_(*t*)_ at trial *t*. Following prior work [5], we treated the unchosen option’s expected value *Q*_*unchosen(t)*_ as a surrogate for the unobserved outcome *R*_*unchosen(t)*_ and computed the context outcome *R*_*CV(t)*_ as:


$$\begin{array}{c} R_{CV(t)}=\;{(R}_{chosen(t)}+Q_{unchosen(t)})/\text{2} \end{array}$$

We used a hierarchical Bayesian approach to estimate each participant’s learning rate (*α*), inverse temperature (*β*), interference sensitivity (*S*), and perseveration bias (*ρ*) [54]. The model comprises three levels: trial-level data, subject-level parameters, and group-level parameters estimated separately for each session-condition combination. Subject-level parameters were assumed to be drawn from group-level distributions defined for each session-condition and estimated using Markov Chain Monte Carlo (MCMC) sampling in PyStan [97]. For each model, we ran 4 Hamiltonian Monte Carlo chains with 1,000 warmup and 1,000 sampling iterations. Model performance was evaluated using the WAIC, which quantifies predictive accuracy, with lower WAIC values indicating better fit [55,56].

In the present study, trials were classified as exploitative if the actual choice matched the model-predicted best option (highest expected rewards or lowest expected punishments) and as exploratory if it did not. This definition—commonly used in previous studies [29,31,38,41–43]—serves as a model-informed behavioral index of exploration rather than a direct estimate of a specific computational parameter [29,35–37,39,40], and does not equate exploration with objectively incorrect choices based on fixed reinforcement probabilities. Exploration rate was defined as the proportion of exploratory choices. In the best-fitting Model 6, this measure was computed after accounting for stochastic choice behavior (*β*), perseveration (*ρ*), and history-dependent interference (*S*). To elucidate the relationship between exploration, reward-learning interference, and loss aversion, we compared exploration rates between RL_INT_ and RL_CON_ trials and examined their associations with the reduction in correct rates in RL_INT_ relative to RL_CON_ trials and with individual loss aversion. In Experiment 3, as reward-learning interference was not statistically significant in the 1 TWD condition (see Results for Experiment 3), exploration rates for the 5 and 25 TWD conditions were pooled.

#### Model validation using simulated data.

As recommended for modeling analyses [57], we performed model recovery on simulated data to validate our model comparison results (S1 Fig). We ran 200 simulations, with each simulation using the number of agents consistent with our experiment. For each model, we simulated datasets using parameter values sampled from the observed range and fit these datasets to all models to determine whether data generated by a given model were best fit (lowest WAIC) by that same model. To further verify that the best-fitting model could recover known parameter values, we conducted parameter recovery by simulating subject-level parameters spanning the empirically observed ranges for the learning rate (*α*), inverse-temperature parameter (*β*), and interference bonus parameter (*S*), and correlating these simulated parameters with those recovered from fitting the model to the simulated data (S2 Fig). To ensure that simulated data from the best-fitting model reproduced key patterns in the observed behavior (Fig 2B and 2C), we conducted posterior predictive checks by simulating behavior from posterior parameter estimates and comparing these simulations with the observed data (S3 Fig). Model convergence was assessed using the Gelman–Rubin *R*-hat statistic, which evaluates convergence across MCMC chains [98]. For all estimated parameters, R-hat values were below 1.02, indicating satisfactory convergence and well-mixed chains.

### Statistical analysis

We used GraphPad Prism software to perform statistical analyses. One-sample *t* tests assessed whether correct rates in each learning condition exceeded chance. For Experiments 1 and 2, self-reported incentive ratings were analyzed via two-way repeated-measures ANOVA to examine the effects of motivation (pursuing rewards or avoiding punishments), punishment type (monetary loss or pain), and their interaction. Task performance was analyzed using three-way repeated-measures ANOVA, assessing learning type (reward or punishment), interference type (control or interference), punishment type, and their interactions. In Experiment 3, incentive ratings were analyzed via a two-way repeated-measures ANOVA for motivation, monetary amount (1, 5, or 25 TWD), and their interaction. Task performance was first analyzed using two-way repeated-measures ANOVA for each monetary amount to assess the effects of learning type, interference type, and their interaction. A one-way repeated-measures ANOVA then examined the effect of monetary amount on the correct rate difference between RL_CON_ and RL_INT_ trials. In Experiment 4, incentive ratings were analyzed using a mixed ANOVA with drug (amisulpride or placebo) as a between-subject factor and motivation and monetary amount (1 or 25 TWD) as within-subject factors. Prolactin levels were examined using a mixed ANOVA with drug and time (baseline or before the learning task) as factors. Fisher’s exact test assessed group differences in exclusion rates. Drug effects on correct and exploration rate differences between RL_CON_ and RL_INT_ trials were analyzed using a 2 (drug) × 2 (monetary amount) mixed ANOVA. Additional mixed ANOVAs tested main and interaction effects of drug and time (baseline, before the learning task, or after the task) on heart rate, blood pressure, mood, and working memory. Pearson’s correlation examined associations between continuous variables. Bonferroni-corrected post hoc tests followed all repeated-measures ANOVAs. Unless otherwise stated, *p* values reflect two-tailed tests. One-tailed tests were used only for analyses testing a priori, theory-driven unidirectional hypotheses, specifically those predicting higher exploration following a history of punishment (PL_CON_ and RL_INT_ versus RL_CON_) and positive associations between exploration, individual loss aversion, and interference-related performance decrements. Because the model specifies a unidirectional mechanism—loss-aversion–driven increases in exploration—effects in the opposite direction are not anticipated within this framework. Sample points (*n*) in all figures represent the number of participants. Results are reported as mean ± SD, with significance set at *p* < 0.05.

### Imaging data acquisition and analysis

Brain images were acquired using a 3-tesla Magnetom Prisma MRI system (Siemens, Erlangen, Germany) equipped with a 64-channel head coil. T2*-weighted BOLD images were collected with a gradient-echo echo planar imaging (EPI) sequence consisting of 37 contiguous axial slices (TR: 2,000 ms; TE: 30 ms; flip angle: 90°; field of view: 224 × 224 mm^2^; GRAPPA acceleration factor: 2; slice thickness: 3.9 mm; voxel size: 3.5 × 3.5 × 3.9 mm^3^; acquisition matrix: 64 × 64). The first four EPI volumes were discarded to ensure steady-state magnetization. For registration, participants underwent high-resolution structural T1-weighted scans using a magnetization-prepared rapid acquisition gradient echo (MP-RAGE) sequence (voxel size: 0.88 × 0.88 × 0.89 mm^3^) and T2-weighted scans coplanar to the EPI with higher in-plane resolution (matrix size: 256 × 256). Magnetic field maps were acquired using a double gradient-echo sequence (TR: 600 ms; TE1: 10.00 ms; TE2: 12.46 ms).

Using SPM12 software (Wellcome Centre for Human Neuroimaging, London, UK), we unwarped echo planar imaging volumes with field maps [99] and corrected for motion artifacts and slice timing differences. The motion-corrected images were then coregistered to the T2-weighted structural images and then aligned with the T1-weighted anatomical images. To improve intersubject registration, the coregistered T1-weighted images were segmented into gray matter, white matter, and cerebrospinal fluid using the International Consortium for Brain Mapping East Asian brain template. Study-specific templates and individual flow fields were generated with the Diffeomorphic Anatomical Registration Through Exponentiated Lie algebra (DARTEL) toolbox. Deformation flow fields for each participant were applied to normalize the realigned functional images to the standard Montreal Neurological Institute space with a 2 × 2 × 2 mm resolution. Spatial smoothing was then performed using a Gaussian kernel with a 6 mm full width at half maximum.

The GLM in SPM12 was employed for fMRI data analysis. For each participant, 3 separate first-level GLMs were established, each including regressors for 3 time points: choice (duration: 2.5 s), post-choice and delay (duration: 3 s), and outcome (duration: 2 s; Fig 1A). To validate learning with related neural activities during our learning task, GLM1 included 6 regressors for the outcome period to model BOLD signals associated with the 6 pairs of options. Trial-by-trial prediction errors—calculated as the difference between the actual outcome and the expected value from the winning Perseveration + Interference at Choice model—were entered as parametric modulators at the time of outcome. This approach allowed us to capture dynamic, trial-specific neural correlates of prediction error beyond the effects of raw outcomes alone. Additional regressors of no interest included 6 regressors for the choice period (one for each pair) and 1 regressor for the post-choice period (collapsed across conditions). Given the critical role of the VS in the processing of prediction errors [46,100] for both appetitive and aversive outcomes [46,47,58–60], here we performed small-volume correction analyses to test whether the bilateral VS encoded prediction error signals pooled across all 6 pairs. Second, to isolate the neural correlate of exploration, GLM2 included 2 regressors for the choice period to model BOLD signals for exploration and exploitation trials, as defined above (see Model specification and fitting). Regressors of no interest included 1 for the post-choice period (collapsed across conditions) and 3 for the outcome period, modeling BOLD signals for monetary gain, monetary loss or pain, and nothing. We conducted whole-brain analyses with the contrast “Exploration> Exploitation” during the choice period to search for activation related to exploration. Third, to examine whether BOLD signals in the dmPFC, identified from GLM2 (see Results), were linked to reward-learning interference, we devised GLM3 in which we set 6 regressors for the choice period (one for each pair). Regressors of no interest included 1 for the post-choice period (collapsed across conditions) and 3 for the outcome period (monetary gain, monetary loss or pain, and nothing). We used the suprathreshold dmPFC cluster from GLM2 to extract BOLD signal for the contrast “RL_INT_> RL_CON_” in GLM3. These extracted signals were then correlated with the reduction in correct rates in RL_INT_ trials relative to RL_CON_ trials. For all GLMs, the 6 motion parameters estimated during realignment were also included as regressors of no interest [101]. All regressors were convolved with a canonical hemodynamic response function [102], and high-pass filtering with a 128 s cutoff was applied to remove low-frequency drifts. First-level *t*-contrasts of interest were subsequently entered into second-level GLM analyses [103].

### ROI definition

To confirm the presence of neural prediction error signals, we defined the ROI for the bilateral VS (two 6-mm-radius spheres centered at MNI coordinates −10/8/−6 and 12/8/−4) according to a prior meta-analysis on appetitive and aversive prediction errors [58].

For group-level whole-brain analyses, we used Statistical non-Parametric Mapping (SnPM13; http://warwick.ac.uk/snpm) to control for false positives from multiple comparisons [104,105]. This approach involved 5,000 permutations without variance smoothing, with a cluster-forming threshold of *p* < 0.001, and a cluster-level familywise error rate of *p* < 0.05 to determine significance. For ROI analyses, small-volume corrections were applied using a familywise error-corrected voxel-wise threshold of *p* < 0.05.

## Supporting information

S1 Fig Model recovery results. To assess whether the experimental design could dissociate the candidate models, we performed model recovery analyses (see Materials and methods, Behavioral analysis and computational modeling of the learning task). In the confusion matrix *P*(fit model | simulated model), each row shows how often a model was selected as best-fitting for data it generated. In the inversion matrix *P*(simulated model | fit model), each row shows the probability that data best fit by a model were actually generated by each model. The confusion matrix was not diagonal for Experiment 1—for example, the Perseveration + Context model was selected as that model only 29% of the time (and as the other two models 38% and 34%). For Experiment 2, diagonality was modest—for example, data generated by the Perseveration model were identified as Perseveration 43% of the time and as Perseveration + Interference at Choice 39% of the time. Despite this, the inversion matrix in both experiments displayed a diagonal tendency: all diagonal entries exceeded 50%, indicating that each model was most likely to be the true generative model when selected as the best-fitting model. Notably, the winning Perseveration + Interference at Choice model was perfectly recoverable (100%) when it served as the generative model in both experiments. When this model was selected as the best-fitting model, it was most likely to correspond to the true generative model (57% in Experiment 1; 62% in Experiment 2), with substantially lower probabilities for alternative models. These results support the use of the best-fitting model in subsequent analyses.
(TIF)

S2 Fig Parameter recovery results for the best-fitting model. To assess the interpretability of the free parameters in the winning Perseveration + Interference at Choice model, we performed parameter recovery using simulated data (see Materials and methods, Behavioral analysis and computational modeling of the learning task). To ensure sufficient coverage of the parameter space and improve the stability of the recovery estimates, simulations were performed using datasets matching the total number of participants across all four experiments. The x-axes show the values used to generate the simulated choices, and the y-axes show the corresponding parameter estimates recovered by fitting the model to those simulations. Pearson correlations between true and recovered parameter values ranged from 0.66 to 0.94, demonstrating good parameter recoverability across the simulated datasets. *α,* learning rate; *β,* inverse temperature parameter; *S*, interference bonus parameter; *ρ*, perseveration bonus parameter.
(TIF)

S3 Fig Posterior predictive simulations for the best-fitting model. To test whether the winning Perseveration + Interference at Choice model captures observed reinforcement learning interference, we performed posterior simulation analyses and visualized simulated behavior. Across both Experiments 1 and 2, the Perseveration + Interference at Choice model (lower panel) more accurately reproduced the reduction in correct rates during reward-learning interference (RL_INT_) relative to reward-learning control (RL_CON_) trials observed in the empirical data than the Perseveration model (upper panel) did, suggesting that interference from the prior punishment history of overlapping options underlies the reward-learning interference effect. The Perseveration model produced no reduction in correct rates during RL_INT_ trials. The participants’ data is shown in circles. The abbreviations in this figure are the same as in Fig 2B and 2C. RL, reward learning; PL, punishment learning. The data underlying this Figure can be found at https://doi.org/10.17605/OSF.IO/T7YWA.
(TIF)

S4 Fig Trial-by-trial learning curves from the best-fitting model. Trial-by-trial changes in correct rate are shown for reward-learning control (RL_CON_; blue solid lines) and reward-learning interference (RL_INT_; blue dashed lines) trials (upper panels), as well as punishment-learning control (PL_CON_; red solid lines) and punishment-learning interference (PL_INT_; red dashed lines) trials (lower panels). Open circles represent observed data, and filled circles denote predictions from the best-fitting Perseveration + Interference at Choice model. Results are displayed for Experiment 1 **(A)** and Experiment 2 **(B)**. The model reproduces the observed behavioral pattern, capturing reduced correct rates in RL_INT_ relative to RL_CON_, while showing no comparable difference between PL_INT_ and PL_CON_.
(TIF)

S5 Fig Model comparison based on baseline-referenced Model 6. The figure shows Watanabe–Akaike Information Criterion (WAIC) differences relative to the baseline-referenced Model 6. Consistent with Model 6 (see Fig 3 for comparison), the baseline-referenced Model 6 outperformed the baseline-referenced Perseveration (Model 5) and Perseveration + Context (Model 11) models, showing the same model ranking pattern. The data underlying this Figure can be found at https://doi.org/10.17605/OSF.IO/T7YWA.
(TIF)

S6 Fig Posterior predictive simulations generated by the baseline-referenced Model 6 across Experiments 1 and 2. Open circles represent observed data, and filled circles denote predictions from the baseline-referenced Model 6. **(A, B)** Consistent with Model 6 (see S3 Fig for comparison), the baseline-referenced Model 6 similarly captured reduced correct rates in reward-learning interference (RL_INT_) relative to reward-learning control (RL_CON_), with no corresponding difference between punishment-learning interference (PL_INT_) and punishment-learning control (PL_CON_). The data underlying this Figure can be found at https://doi.org/10.17605/OSF.IO/T7YWA. **(C, D)** Consistent with Model 6 (see S4 Fig for comparison), it also reproduced the trial-by-trial pattern of behavior, showing lower correct rates in RL_INT_ relative to RL_CON_ but no comparable difference between PL_INT_ and PL_CON_.
(TIF)

S7 FigPosterior distributions of condition differences in group-level parameter means (*μ*) in baseline-referenced Model 6.Shown are posterior distributions of group-level mean estimates (*μ*) for the interference parameter (*S*), perseveration parameter (*ρ*), and the natural logarithm of the inverse temperature parameter (*β*) across sessions and experiments in the baseline-referenced Model 6. Blue solid lines indicate reward-learning control (RL_CON_) trials, and blue dashed lines indicate reward-learning interference (RL_INT_; i.e., RL_CON_ + $\Delta S_{{RL}_{INT}}$) trials. Vertical black lines denote posterior medians, and shaded regions indicate 95% highest density intervals (HDIs). See S3 Table for posterior means, SDs, and HDI ranges.(TIF)

S8 Fig Relationship between reward-learning interference and exploration based on baseline-referenced Model 6. **(A, B)** In Experiment 1, exploration rates were higher in PL_CON_ than RL_CON_ trials (*p* = 0.001, one-tailed), and also elevated in RL_INT_ trials (*p* = 0.037, one-tailed). Increased exploration in RL_INT_ relative to RL_CON_ correlated with reduced correct responses (*p* = 0.036, one-tailed), indicating a link between exploration and reward-learning interference. **(C, D)** Experiment 2 showed a similar pattern, with exploration differences across conditions and a significant correlation between RL_INT_–RL_CON_ exploration increase and reduced accuracy (*p* = 0.012). **(E, F)** In Experiment 3, PL_CON_ consistently showed higher exploration than RL_CON_ across monetary conditions (all *p* ≤ 0.004). RL_INT_ exceeded RL_CON_ in the 5 and 25 TWD conditions (both *p* ≤ 0.002; not significant in 1 TWD), and RL_INT_-related increases in exploration correlated with reduced accuracy (*p* = 0.019, one-tailed; pooled 5 and 25 TWD). These results are similar to those based on Model 6 (see Fig 4 for comparison). In (A), (C), and (E), *p* values are Bonferroni-corrected from repeated-measures ANOVAs. Data are mean ± SD. The data underlying S8A, S8C, and S8E Fig can be found at https://doi.org/10.17605/OSF.IO/T7YWA.
(TIF)

S9 Fig Monetary amount-dependent reward-learning interference, exploration, and its association with loss aversion in Experiment 3 based on baseline-referenced Model 6. **(A)** Reward-learning interference (reduced correct rate in RL_INT_ versus RL_CON_) increased with monetary amount, with a significant difference between 1 and 25 TWD (*p* = 0.036). **(B)** RL_INT_–RL_CON_ exploration differences showed the same pattern, also significant between 1 and 25 TWD (*p* = 0.024). (C) RL_INT_–RL_CON_ increases in exploration correlated with loss aversion (pooled 5 and 25 TWD; *p* = 0.023, one-tailed). These results are similar to those based on Model 6 (see Fig 6 for comparison). All *p* values in (A) and (B) are Bonferroni-corrected in repeated-measures ANOVAs. Data are represented as mean ± SD. The data underlying S9A and S9B Fig can be found at https://doi.org/10.17605/OSF.IO/T7YWA.
(TIF)

S10 Fig Effect of dopamine antagonist treatment on exploration and reward-learning interference in Experiment 4 based on baseline-referenced Model 6. **(A)** Treatment modulated reward-learning interference (RL_INT_ versus RL_CON_ correct rates) across monetary amounts (1 versus 25 TWD; *p* = 0.019). In the placebo group, interference was larger at 25 than 1 TWD (*p* = 0.004), whereas no difference was observed under amisulpride (*p* > 0.999). (B) A similar interaction was observed for RL_INT_–RL_CON_ exploration differences (*p* = 0.017), with a 25 > 1 TWD effect in placebo (*p* = 0.033) but not in the amisulpride group (*p* = 0.595). These results are similar to those based on Model 6 (see Fig 7 for comparison). All *p* values correspond to interaction effects in mixed ANOVAs. Data are represented as mean ± SD. The data underlying this Figure can be found at https://doi.org/10.17605/OSF.IO/T7YWA.
(TIF)

S11 Fig Neural prediction error signals. **(A)** Trial-by-trial prediction error signals were observed in the bilateral ventral striatum (VS) during the learning task in Experiment 1. **(B)** Similar signals were observed in Experiment 2, with both experiments modeled using general linear model (GLM) 1 (see Materials and methods). All activated clusters are voxel-wise small-volume family-wise error corrected with a threshold of *p* < 0.05. To accurately present small-volume correction results, only suprathreshold voxels are illustrated.
(TIF)

S1 Table Choice behavior. The table summarizes (i) task performance (correct rate) in reward-learning interference (RL_INT_) and punishment-learning interference (PL_INT_) trials, along with their corresponding control trials (RL_CON_ and PL_CON_), and (ii) choice rates for option *E* (*EF* pair) and option *G* (*GH* pair). In Experiments 1 and 2, a 2 (learning type: reward versus punishment) × 2 (interference type: control versus interference) × 2 (punishment type: monetary loss versus pain) repeated-measures ANOVA revealed a significant main effect of interference type (both *p* < 0.001) and a significant learning-by-interference interaction (^#^ *p* < 0.05), reflecting reduced performance in RL_INT_ relative to RL_CON_ trials, while no reliable differences were observed between PL_INT_ and PL_CON_ trials. No significant main effect of learning type or punishment type (both *p* ≥ 0.144), nor a three-way interaction (*p* = 0.701), was observed, indicating no significant difference between the two punishment types in their interference effects. In Experiments 3 and 4, interference effects were evaluated using a 2 (learning type) × 2 (interference type) repeated-measures ANOVA (^#^ *p* < 0.05). Across experiments, punishment was implemented as either monetary loss (1, 5, or 25 New Taiwan Dollars) or painful stimulation. Data are shown as mean ± SD. ^*^ *p* < 0.05 compared with chance level (0.5; one-sample *t* test). See Results for details.
(XLSX)

S2 Table Watanabe-Akaike information Criterion (WAIC) values. This table shows the WAIC values for the 19 computational models across all experiments. Lower WAIC values indicate better model fit. Across all four experiments, the Perseveration + Interference at Choice model best accounted for the behavioral data, yielding the lowest WAIC in each experiment. Ami, amisulpride group in Experiment 4; CV, contextual value; DL, delta-learning rule; IT, an interference term applied to the choice phase, learning rate (LR), or prediction error (PE); Pla, placebo group in Experiment 4; PT, perseveration term; SM, softmax function.
(XLSX)

S3 Table Posterior estimates of condition differences in group-level parameter means (*μ*) in the baseline-referenced Model 6. The table reports posterior estimates of condition differences (RL_INT_ − RL_CON_) in group-level parameter means for each experiment and session, including posterior means (standard deviations in parentheses), 95% highest density intervals (HDIs), and posterior probabilities that the difference between RL_INT_ and RL_CON_ is greater or less than zero. In the baseline-referenced Model 6, RL_CON_ serves as the reference baseline, and Δ parameters represent condition-specific deviations from this baseline (see Methods). In Experiments 1 and 2, punishment in Sessions 1–2 involved monetary loss, whereas Sessions 3–4 involved pain. In Experiment 3, punishment in Sessions 1–3 involved monetary losses of 1, 5, and 25 New Taiwan dollars, respectively. *S*: interference parameter; *β*: inverse temperature parameter; *ρ*: perseveration parameter.
(XLSX)

S4 Table Exploration rate. This table shows exploration rates in reward-learning interference (RL_INT_) and punishment-learning interference (PL_INT_) trials, along with their corresponding control trials (RL_CON_ and PL_CON_) across all four experiments. ^*^ *p* < 0.05 (paired *t* test, one-tailed). In each experiment, the punishment associated with each option was either a monetary loss (1, 5, or 25 New Taiwan Dollars) or a painful stimulus. Comparisons between PL_INT_ and PL_CON_ trials did not reach statistical significance in any experiment (all *p* ≥ 0.07) and are therefore not listed. Data are represented as mean ± SD. See Results for details.
(XLSX)

S5 Table Whole-brain activations related to exploration. This table shows peak coordinates, *t* value, and activated cluster size (in voxels in parentheses) of activated brain regions of whole-brain analyses using SnPM (family-wise error correction at a cluster-level of *p* < 0.05) during the 2.5 s choice period. In Experiment 1, activated clusters are numbered (e.g., contrast “Exploration> Exploitation” produced four activated clusters, whose sizes are 394, 133, and 208 voxels), and activation foci corresponding to different anatomical locations within the clusters are given as MNI coordinates (in millimeters). aIC, anterior insular cortex; IFG, inferior frontal gyrus; L, left; MCC, middle cingulate cortex; PAG, periaqueductal gray; R, right; SMA, supplementary motor area.
(XLSX)

## Abbreviations

CV: context value
dmPFC: dorsomedial prefrontal cortex
EPI: echo planar imaging
GLM: general linear model
MCMC: Markov Chain Monte Carlo
MP-RAGE: magnetization-prepared rapid acquisition gradient echo
VS: ventral striatum
WAIC: Watanabe–Akaike Information Criterion
