# Supplementary figures and images for: Competing value signals impair reward-learning via dopaminergic mechanisms and increase exploration
Source: PLoS Biol. 2026 Jul 24;24(7):e3003922. doi: 10.1371/journal.pbio.3003922 (PMC13427004; doi:10.1371/journal.pbio.3003922)

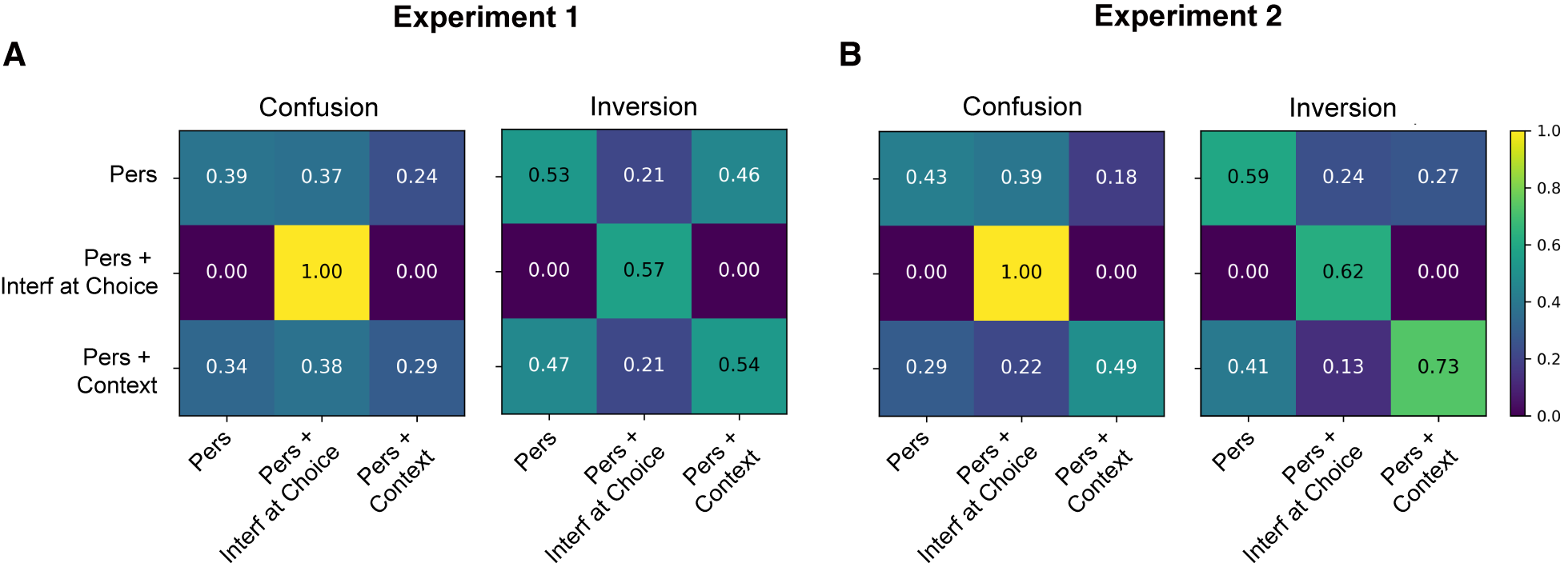

Supplement: S1 Fig — To assess whether the experimental design could dissociate the candidate models, we performed model recovery analyses (see Materials and methods, Behavioral analysis and computational modeling of the learning task). In the confusion matrix P(fit model | simulated model), each row shows how often a model was selected as best-fitting for data it generated. In the inversion matrix P(simulated model | fit model), each row shows the probability that data best fit by a model were actually generated by each model. The confusion matrix was not diagonal for Experiment 1—for example, the Perseveration + Context model was selected as that model only 29% of the time (and as the other two models 38% and 34%). For Experiment 2, diagonality was modest—for example, data generated by the Perseveration model were identified as Perseveration 43% of the time and as Perseveration + Interference at Choice 39% of the time. Despite this, the inversion matrix in both experiments displayed a diagonal tendency: all diagonal entries exceeded 50%, indicating that each model was most likely to be the true generative model when selected as the best-fitting model. Notably, the winning Perseveration + Interference at Choice model was perfectly recoverable (100%) when it served as the generative model in both experiments. When this model was selected as the best-fitting model, it was most likely to correspond to the true generative model (57% in Experiment 1; 62% in Experiment 2), with substantially lower probabilities for alternative models. These results support the use of the best-fitting model in subsequent analyses. (TIF) [file pbio.3003922.s001.tif]

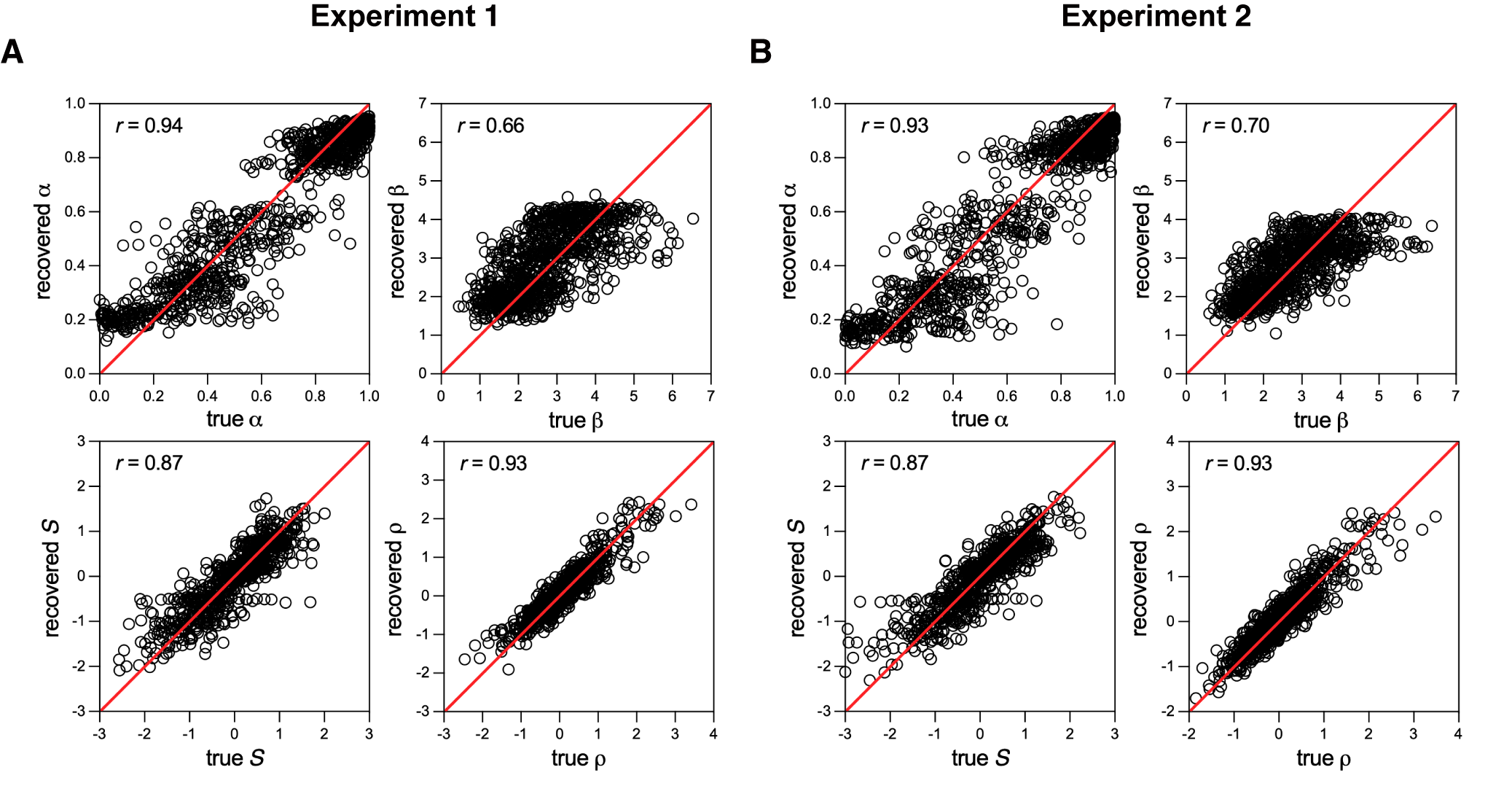

Supplement: S2 Fig — To assess the interpretability of the free parameters in the winning Perseveration + Interference at Choice model, we performed parameter recovery using simulated data (see Materials and methods, Behavioral analysis and computational modeling of the learning task). To ensure sufficient coverage of the parameter space and improve the stability of the recovery estimates, simulations were performed using datasets matching the total number of participants across all four experiments. The x-axes show the values used to generate the simulated choices, and the y-axes show the corresponding parameter estimates recovered by fitting the model to those simulations. Pearson correlations between true and recovered parameter values ranged from 0.66 to 0.94, demonstrating good parameter recoverability across the simulated datasets. α, learning rate; β, inverse temperature parameter; S, interference bonus parameter; ρ, perseveration bonus parameter. (TIF) [file pbio.3003922.s002.tif]

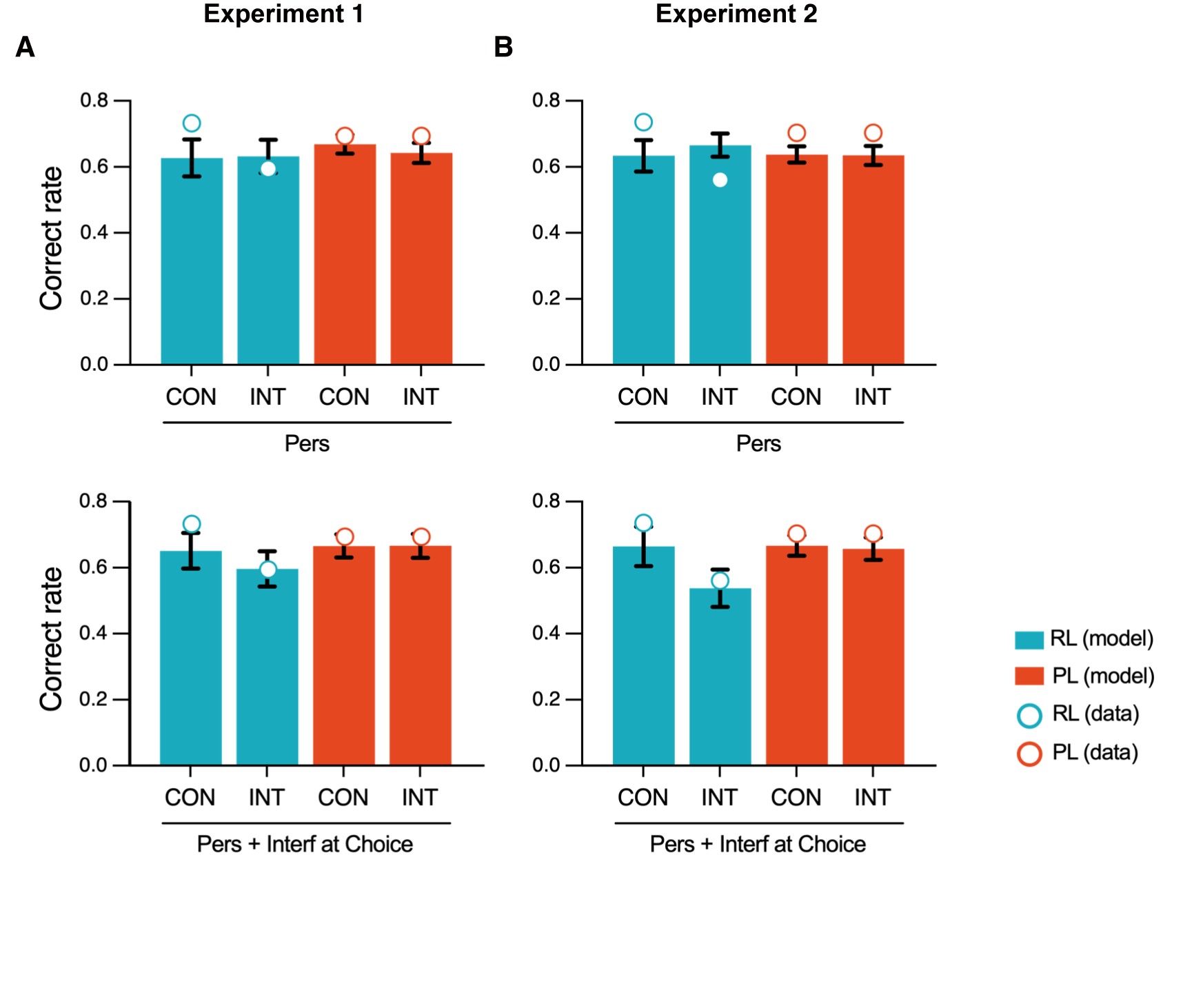

Supplement: S3 Fig — To test whether the winning Perseveration + Interference at Choice model captures observed reinforcement learning interference, we performed posterior simulation analyses and visualized simulated behavior. Across both Experiments 1 and 2, the Perseveration + Interference at Choice model (lower panel) more accurately reproduced the reduction in correct rates during reward-learning interference (RLINT) relative to reward-learning control (RLCON) trials observed in the empirical data than the Perseveration model (upper panel) did, suggesting that interference from the prior punishment history of overlapping options underlies the reward-learning interference effect. The Perseveration model produced no reduction in correct rates during RLINT trials. The participants’ data is shown in circles. The abbreviations in this figure are the same as in Fig 2B and 2C. RL, reward learning; PL, punishment learning. The data underlying this Figure can be found at https://doi.org/10.17605/OSF.IO/T7YWA. (TIF) [file pbio.3003922.s003.tif]

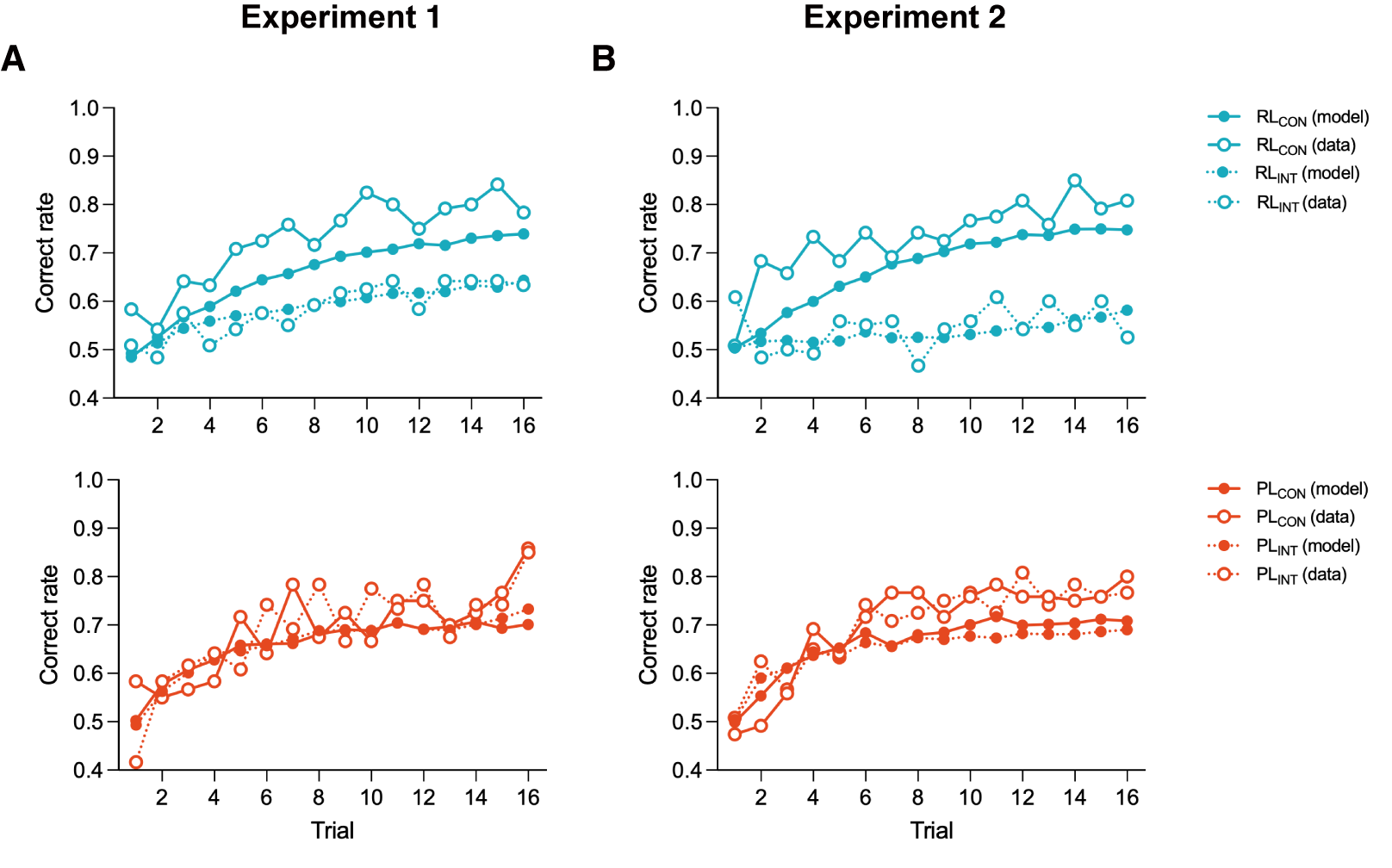

Supplement: S4 Fig — Trial-by-trial changes in correct rate are shown for reward-learning control (RLCON; blue solid lines) and reward-learning interference (RLINT; blue dashed lines) trials (upper panels), as well as punishment-learning control (PLCON; red solid lines) and punishment-learning interference (PLINT; red dashed lines) trials (lower panels). Open circles represent observed data, and filled circles denote predictions from the best-fitting Perseveration + Interference at Choice model. Results are displayed for Experiment 1 (A) and Experiment 2 (B). The model reproduces the observed behavioral pattern, capturing reduced correct rates in RLINT relative to RLCON, while showing no comparable difference between PLINT and PLCON. (TIF) [file pbio.3003922.s004.tif]

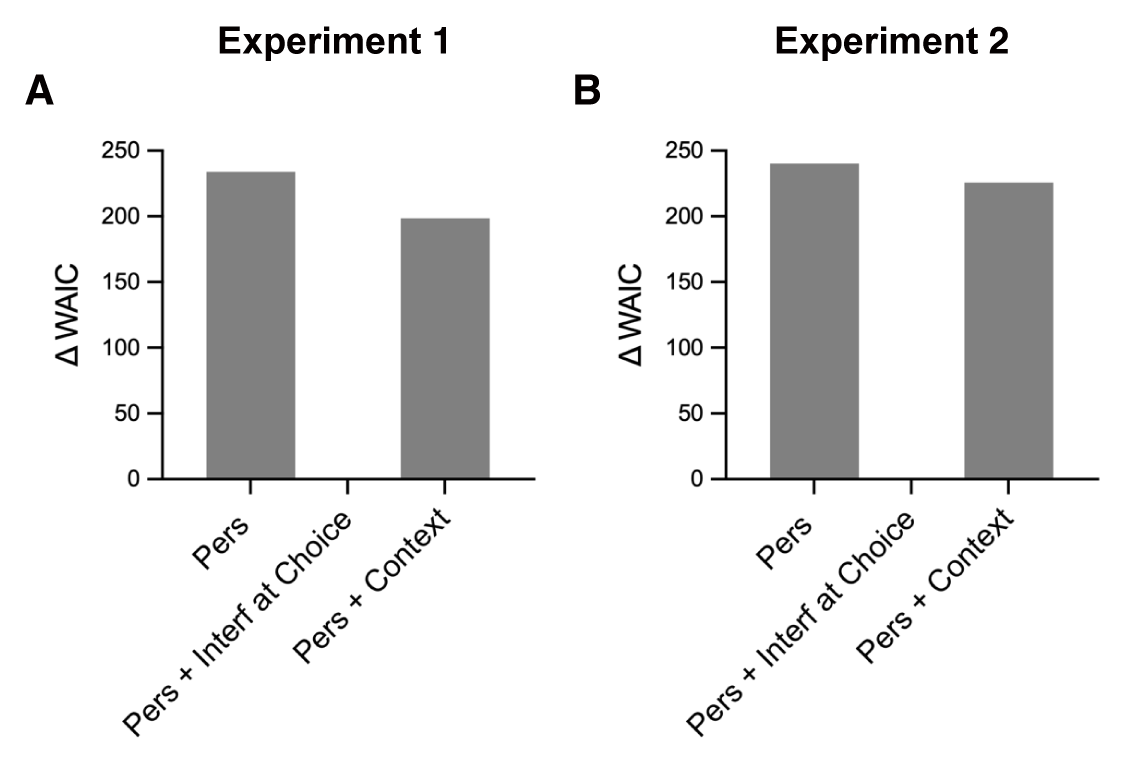

Supplement: S5 Fig — The figure shows Watanabe–Akaike Information Criterion (WAIC) differences relative to the baseline-referenced Model 6. Consistent with Model 6 (see Fig 3 for comparison), the baseline-referenced Model 6 outperformed the baseline-referenced Perseveration (Model 5) and Perseveration + Context (Model 11) models, showing the same model ranking pattern. The data underlying this Figure can be found at https://doi.org/10.17605/OSF.IO/T7YWA. (TIF) [file pbio.3003922.s005.tif]

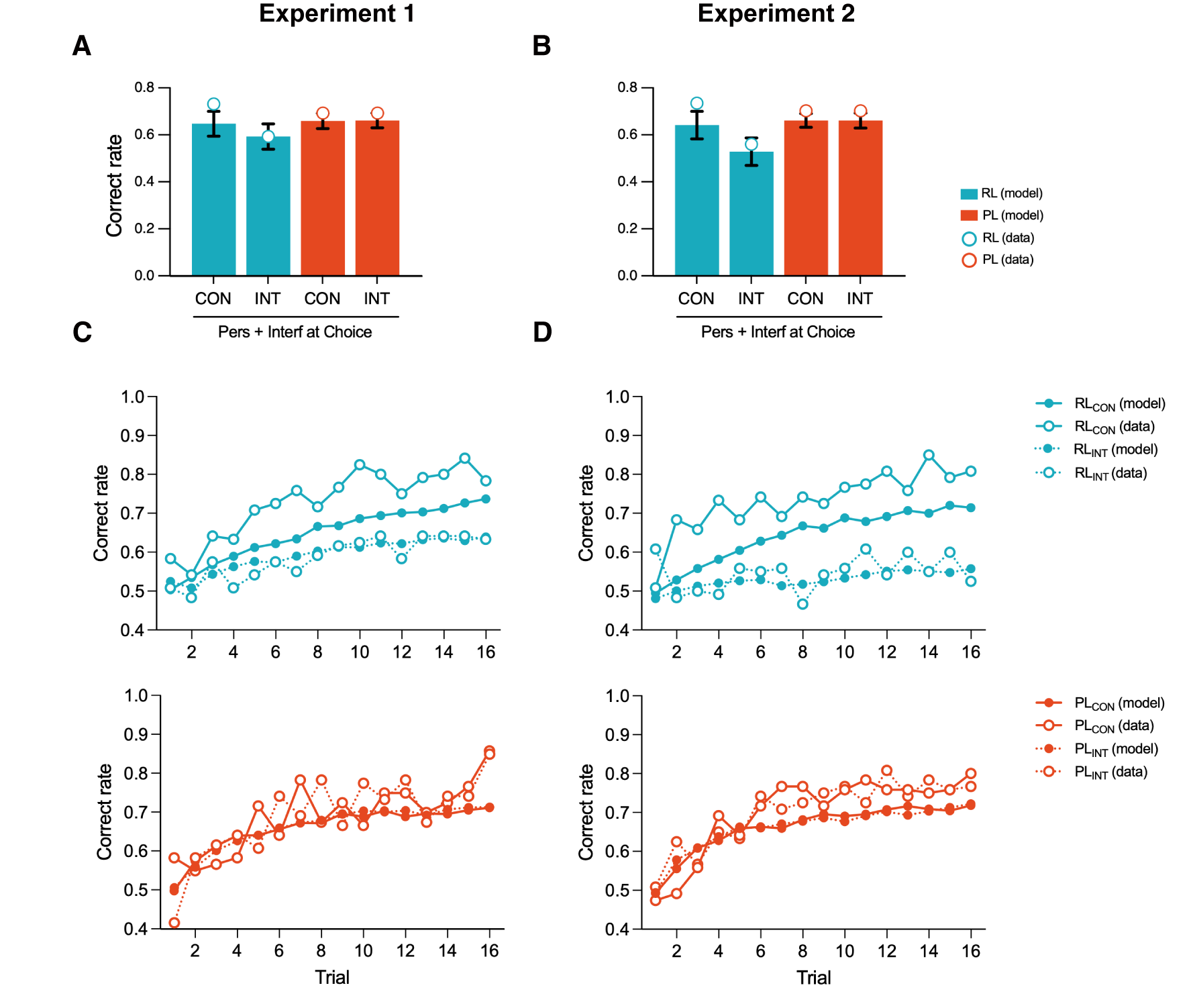

Supplement: S6 Fig — Open circles represent observed data, and filled circles denote predictions from the baseline-referenced Model 6. (A, B) Consistent with Model 6 (see S3 Fig for comparison), the baseline-referenced Model 6 similarly captured reduced correct rates in reward-learning interference (RLINT) relative to reward-learning control (RLCON), with no corresponding difference between punishment-learning interference (PLINT) and punishment-learning control (PLCON). The data underlying this Figure can be found at https://doi.org/10.17605/OSF.IO/T7YWA. (C, D) Consistent with Model 6 (see S4 Fig for comparison), it also reproduced the trial-by-trial pattern of behavior, showing lower correct rates in RLINT relative to RLCON but no comparable difference between PLINT and PLCON. (TIF) [file pbio.3003922.s006.tif]

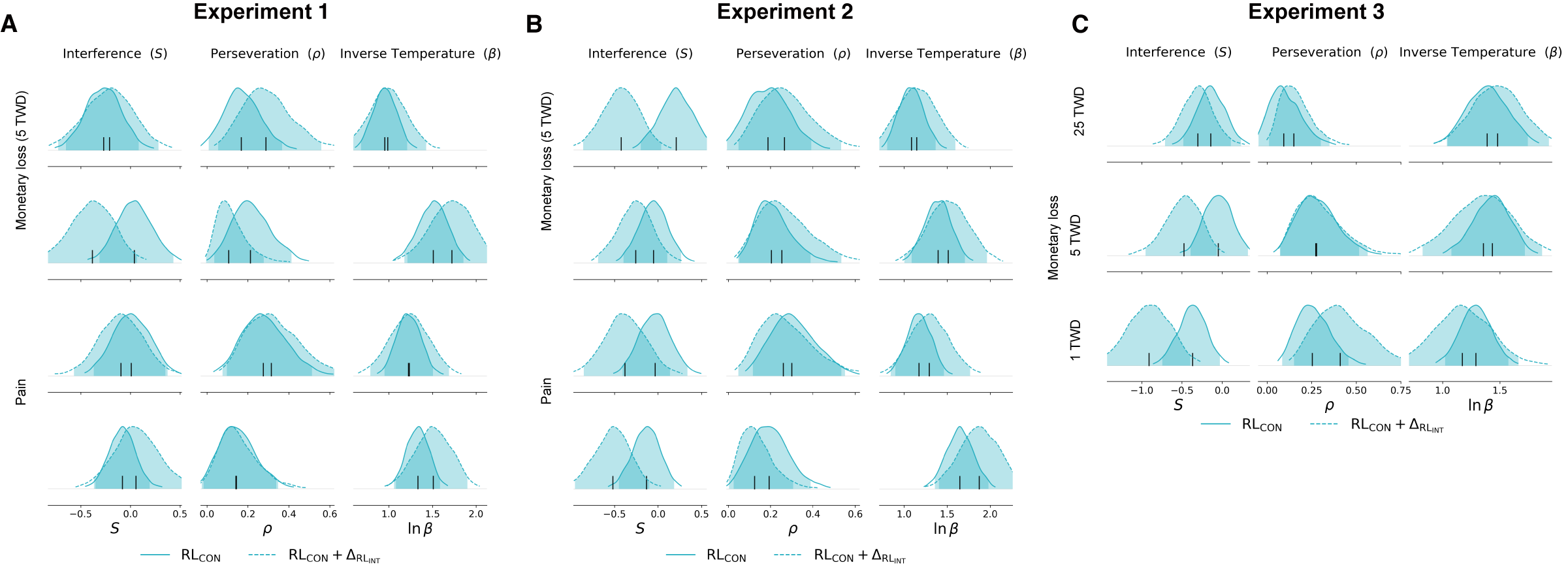

Supplement: S7 Fig — Shown are posterior distributions of group-level mean estimates (μ) for the interference parameter (S), perseveration parameter (ρ), and the natural logarithm of the inverse temperature parameter (β) across sessions and experiments in the baseline-referenced Model 6. Blue solid lines indicate reward-learning control (RLCON) trials, and blue dashed lines indicate reward-learning interference (RLINT; i.e., RLCON + ΔSRLINT) trials. Vertical black lines denote posterior medians, and shaded regions indicate 95% highest density intervals (HDIs). See S3 Table for posterior means, SDs, and HDI ranges. (TIF) [file pbio.3003922.s007.tif]

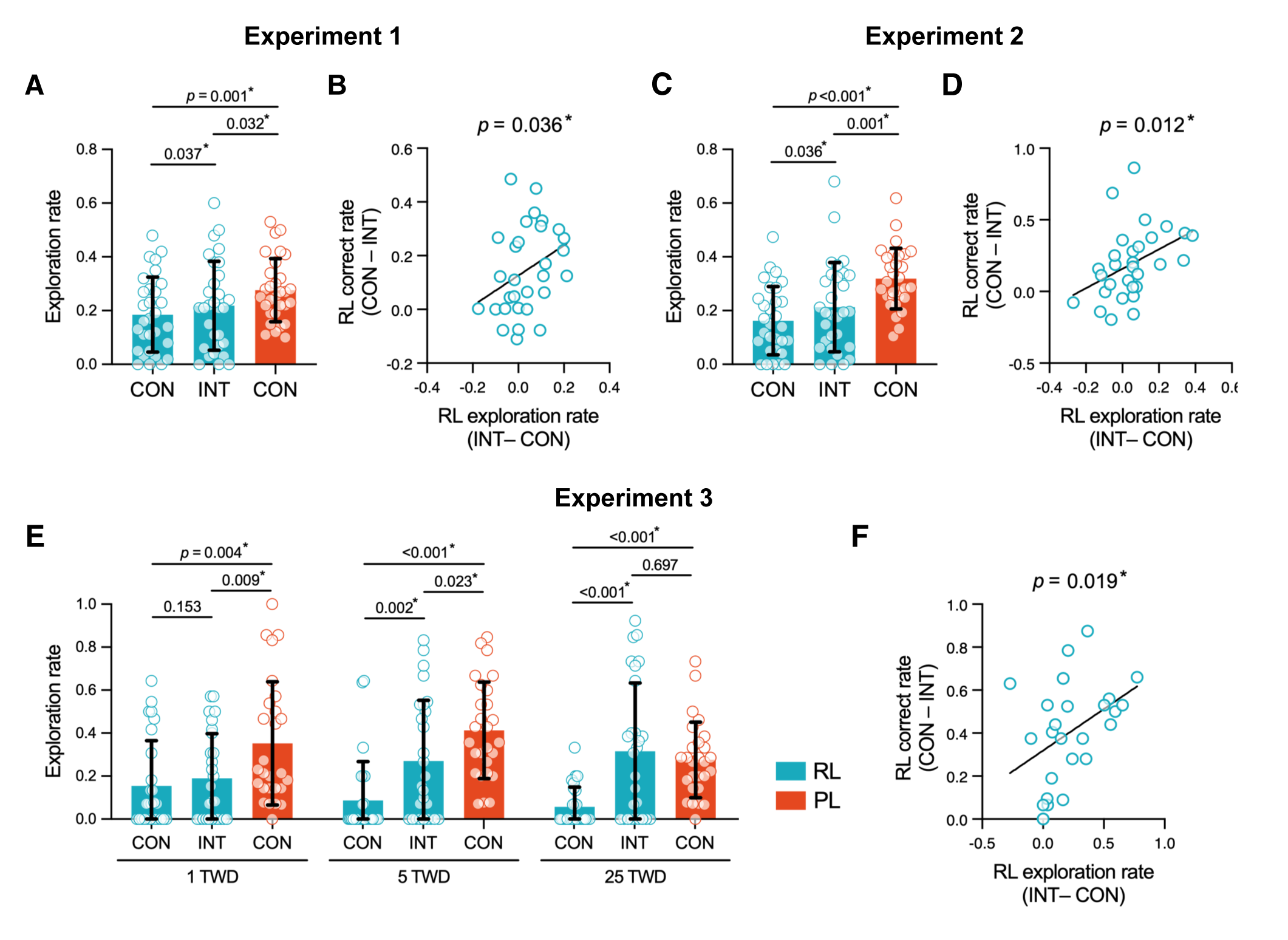

Supplement: S8 Fig — (A, B) In Experiment 1, exploration rates were higher in PLCON than RLCON trials (p = 0.001, one-tailed), and also elevated in RLINT trials (p = 0.037, one-tailed). Increased exploration in RLINT relative to RLCON correlated with reduced correct responses (p = 0.036, one-tailed), indicating a link between exploration and reward-learning interference. (C, D) Experiment 2 showed a similar pattern, with exploration differences across conditions and a significant correlation between RLINT–RLCON exploration increase and reduced accuracy (p = 0.012). (E, F) In Experiment 3, PLCON consistently showed higher exploration than RLCON across monetary conditions (all p ≤ 0.004). RLINT exceeded RLCON in the 5 and 25 TWD conditions (both p ≤ 0.002; not significant in 1 TWD), and RLINT-related increases in exploration correlated with reduced accuracy (p = 0.019, one-tailed; pooled 5 and 25 TWD). These results are similar to those based on Model 6 (see Fig 4 for comparison). In (A), (C), and (E), p values are Bonferroni-corrected from repeated-measures ANOVAs. Data are mean ± SD. The data underlying S8A, S8C, and S8E Fig can be found at https://doi.org/10.17605/OSF.IO/T7YWA. (TIF) [file pbio.3003922.s008.tif]

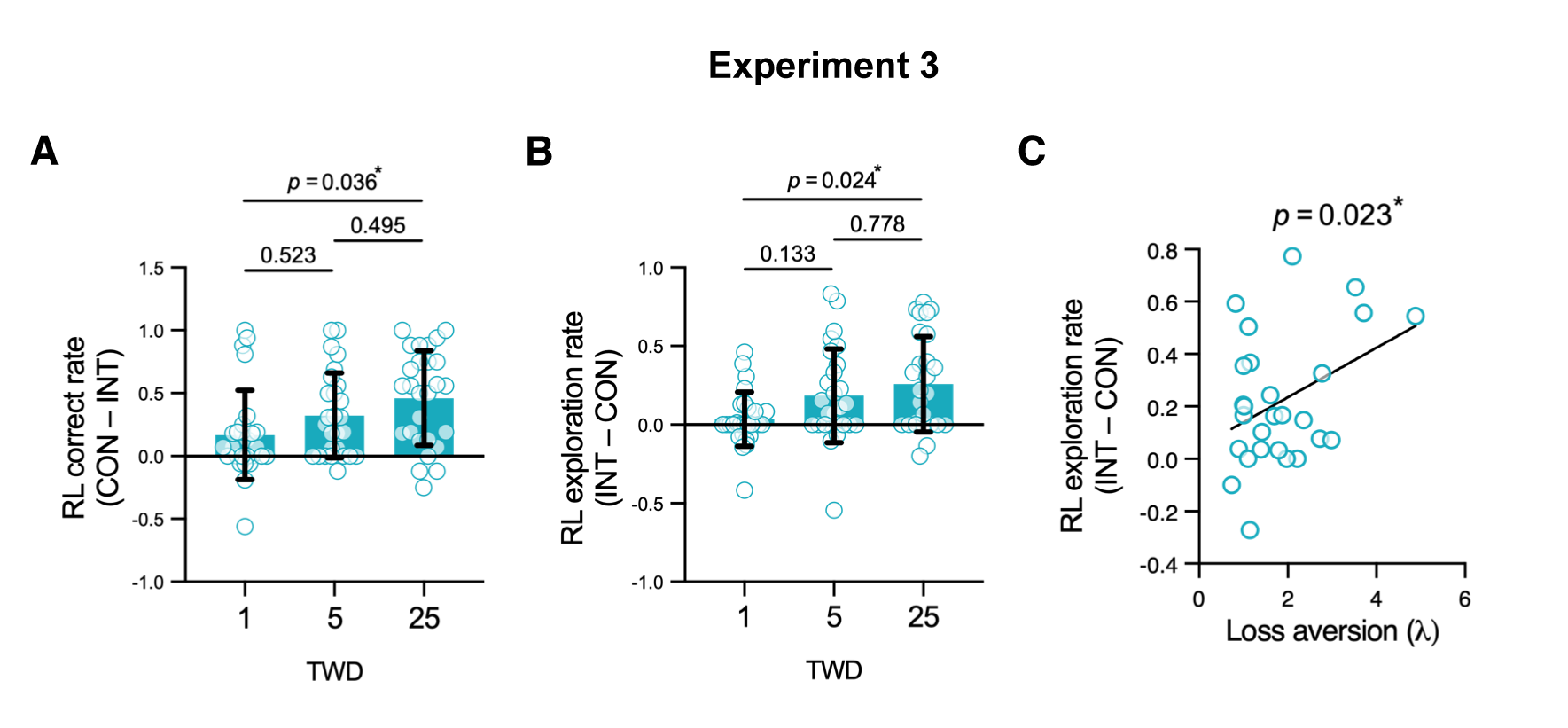

Supplement: S9 Fig — (A) Reward-learning interference (reduced correct rate in RLINT versus RLCON) increased with monetary amount, with a significant difference between 1 and 25 TWD (p = 0.036). (B) RLINT–RLCON exploration differences showed the same pattern, also significant between 1 and 25 TWD (p = 0.024). (C) RLINT–RLCON increases in exploration correlated with loss aversion (pooled 5 and 25 TWD; p = 0.023, one-tailed). These results are similar to those based on Model 6 (see Fig 6 for comparison). All p values in (A) and (B) are Bonferroni-corrected in repeated-measures ANOVAs. Data are represented as mean ± SD. The data underlying S9A and S9B Fig can be found at https://doi.org/10.17605/OSF.IO/T7YWA. (TIF) [file pbio.3003922.s009.tif]

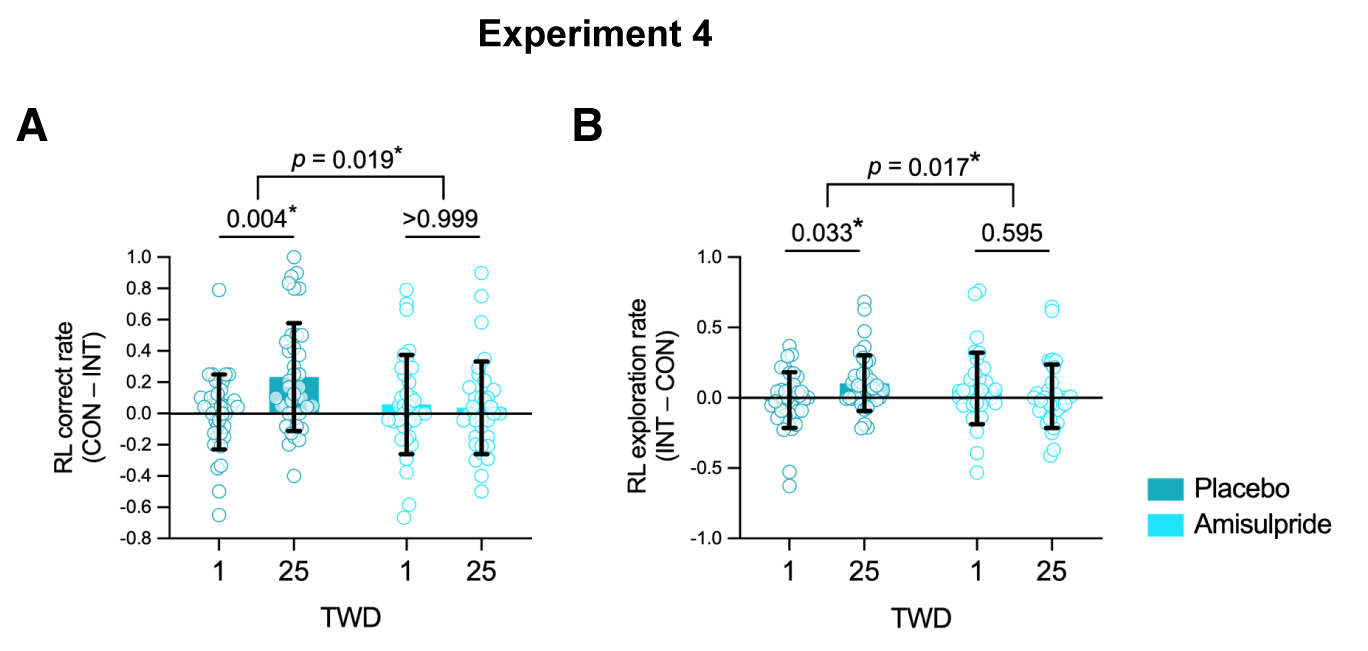

Supplement: S10 Fig — (A) Treatment modulated reward-learning interference (RLINT versus RLCON correct rates) across monetary amounts (1 versus 25 TWD; p = 0.019). In the placebo group, interference was larger at 25 than 1 TWD (p = 0.004), whereas no difference was observed under amisulpride (p > 0.999). (B) A similar interaction was observed for RLINT–RLCON exploration differences (p = 0.017), with a 25 > 1 TWD effect in placebo (p = 0.033) but not in the amisulpride group (p = 0.595). These results are similar to those based on Model 6 (see Fig 7 for comparison). All p values correspond to interaction effects in mixed ANOVAs. Data are represented as mean ± SD. The data underlying this Figure can be found at https://doi.org/10.17605/OSF.IO/T7YWA. (TIF) [file pbio.3003922.s010.tif]

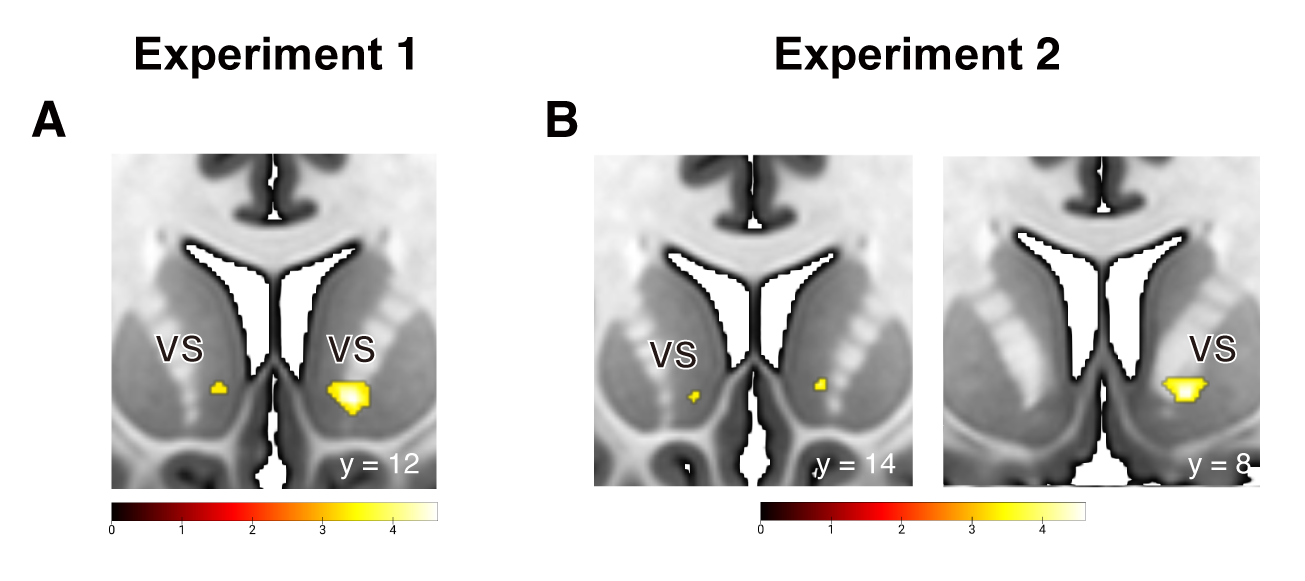

Supplement: S11 Fig — (A) Trial-by-trial prediction error signals were observed in the bilateral ventral striatum (VS) during the learning task in Experiment 1. (B) Similar signals were observed in Experiment 2, with both experiments modeled using general linear model (GLM) 1 (see Materials and methods). All activated clusters are voxel-wise small-volume family-wise error corrected with a threshold of p < 0.05. To accurately present small-volume correction results, only suprathreshold voxels are illustrated. (TIF) [file pbio.3003922.s011.tif]
